# Endemic, exotic and novel apicomplexan parasites detected during a national study of ticks from companion animals in Australia

**DOI:** 10.1186/s13071-018-2775-y

**Published:** 2018-03-20

**Authors:** Telleasha L. Greay, Alireza Zahedi, Anna-Sheree Krige, Jadyn M. Owens, Robert L. Rees, Una M. Ryan, Charlotte L. Oskam, Peter J. Irwin

**Affiliations:** 1 Vector and Waterborne Pathogens Research Group, School of Veterinary and Life Sciences, Murdoch University, Perth, Western Australia Australia; 2 Western Australian State Agricultural Biotechnology Centre, Murdoch University, Perth, Western Australia Australia; 3 Faculty of Veterinary and Agricultural Sciences, The University of Melbourne, Melbourne, Victoria Australia

**Keywords:** Ticks, New species, Dogs, Cats, Horses, *18S* ribosomal RNA gene, Apicomplexa, *Babesia*, *Theileria*, *Hepatozoon*, Sarcocystidae

## Abstract

**Background:**

Apicomplexan tick-borne pathogens that cause disease in companion animals include species of *Babesia* Starcovici, 1893, *Cytauxzoon* Neitz & Thomas, 1948, *Hepatozoon* Miller, 1908 and *Theileria* Bettencourt, Franca & Borges, 1907. The only apicomplexan tick-borne disease of companion animals that is known to occur in Australia is babesiosis, caused by *Babesia canis vogeli* Reichenow, 1937 and *Babesia gibsoni* Patton, 1910*.* However, no molecular investigations have widely investigated members of Apicomplexa Levine, 1980 in Australian ticks that parasitise dogs, cats or horses, until this present investigation.

**Results:**

Ticks (*n* = 711) removed from dogs (*n* = 498), cats (*n* = 139) and horses (*n* = 74) throughout Australia were screened for piroplasms and *Hepatozoon* spp. using conventional PCR and Sanger sequencing. The tick-borne pathogen *B. vogeli* was identified in two *Rhipicephalus sanguineus* Latreille ticks from dogs residing in the Northern Territory and Queensland (QLD). *Theileria orientalis* Yakimov & Sudachenkov, 1931 genotype Ikeda was detected in three *Haemaphysalis longicornis* Neumann ticks from dogs in New South Wales. Unexpectedly, the exotic tick-borne pathogen *Hepatozoon canis* James, 1905 was identified in an *Ixodes holocyclus* Neumann tick from a dog in QLD. Eight novel piroplasm and *Hepatozoon* species were identified and described in native ticks and named as follows: *Babesia lohae* n. sp., *Babesia mackerrasorum* n. sp., *Hepatozoon banethi* n. sp., *Hepatozoon ewingi* n. sp., *Theileria apogeana* n. sp., *Theileria palmeri* n. sp., *Theileria paparinii* n. sp. and *Theileria worthingtonorum* n. sp. Additionally, a novel cf. Sarcocystidae sp. sequence was obtained from *Ixodes tasmani* Neumann but could not be confidently identified at the genus level.

**Conclusions:**

Novel species of parasites in ticks represent an unknown threat to the health of companion animals that are bitten by these native tick species. The vector potential of Australian ticks for the newly discovered apicomplexans needs to be assessed, and further clinical and molecular investigations of these parasites, particularly in blood samples from dogs, cats and horses, is required to determine their potential for pathogenicity.

**Electronic supplementary material:**

The online version of this article (10.1186/s13071-018-2775-y) contains supplementary material, which is available to authorized users.

## Background

Intracellular apicomplexan parasites consist of the groups haemococcidia, haemogregarines, haemosporidia and piroplasms; collectively these haemoprotozoa are transmitted by haematophagous vectors, such as ticks (Acari: Ixodida). Apicomplexan tick-borne diseases of companion animals are caused by the piroplasms *Babesia* spp., *Cytauxzoon felis* Kier, 1979 and *Theileria* spp., and the haemogregarines *Hepatozoon* Miller, 1908 spp. Unlike other developed countries with high pet ownership rates, such as the USA [1], relatively few apicomplexan tick-borne pathogens have been reported to affect pets in Australia; these include *Babesia canis vogeli* Reichenow, 1937 [2, 3] and *Babesia gibsoni* Patton, 1910 [4], which cause babesiosis in domestic dogs (*Canis lupus familiaris* Linnaeus). The brown dog tick (*Rhipicephalus sanguineus* Latreille), a species that was introduced into the Australian continent in relatively recent times, is the vector of *B. vogeli* [5, 6] and a putative vector of *B. gibsoni* [4]. Although the majority of ticks collected from dogs during a 2012–2015 national tick survey in Australia were identified as *R. sanguineus* (73%) [7], seven out of the ten tick species identified on dogs were native ticks that, to the best of our knowledge, have not been widely investigated for their association with apicomplexan parasites previously. Similarly, most of the tick species that were identified on horses (*Equus ferus caballus* Linnaeus), and all of those removed from cats (*Felis catus* Linnaeus), were also native ticks [7]. To gain a more comprehensive understanding of the potentially vector-borne parasites associated with Australian ticks, the current study aimed to screen ticks that were parasitising dogs, cats and horses for piroplasms and *Hepatozoon* spp. using conventional PCR and Sanger sequencing.

## Methods

### Tick collection and identification

Ticks were opportunistically collected during a nationwide tick survey between 2012–2015 (*n* = 4765) [7]. Individual specimens were removed from companion animals, stored in 70% ethanol and morphologically identified [8, 9]. A subset of ticks (*n* = 711) was selected for the present study from dogs (*n* = 498), cats (*n* = 139) and horses (*n* = 74) (Table 1). Collection locations included all Australian states and territories, except for the Australian Capital Territory. A summary of the tick collection locations is provided in Additional file 1: Table S1.

**Table 1:** Summary of ticks sampled from dogs, cats and horses for piroplasm and *Hepatozoon* spp. screening

Tick species	Dogs	Cats	Horses	Total
*Amblyomma triguttatum triguttatum*	11	0	13	24
*Bothriocroton* cf. *auruginans*^a^	6	0	0	6
*Haemaphysalis bancrofti*	1	1	3	5
*Haemaphysalis lagostrophi*	0	0	1	1
*Haemaphysalis longicornis*	51	0	24	75
Ixodida: Ixodidae, cf. *Haemaphysalis*^b^	0	0	3	3
*Ixodes cornuatus*	9	1	0	10
*Ixodes hirsti*	0	1	0	1
*Ixodes holocyclus*	182	126	27	335
*Ixodes myrmecobii*	4	1	0	5
*Ixodes tasmani*	49	9	1	59
*Rhipicephalus australis*	1	0	2	3
*Rhipicephalus sanguineus*	184	0	0	184
Grand total	498	139	74	711

^a^Adult ticks were too damaged to allow for morphological identification and many of the immature life stages of *Bothriocroton* spp. have not yet been described

^b^These specimens did not match morphological descriptions for *Haemaphysalis* spp. in the Australian tick identification keys [8, 9]

### DNA extractions

Tick specimens were bisected and genomic DNA (gDNA) was extracted from one half of the tick (cut into smaller pieces with sterile scalpel blades; the other half of the specimen was stored in ethanol for future studies) using a DNeasy Blood & Tissue Kit (Qiagen, Hilden, Germany) following the manufacturer’s recommendations, with the following modifications: after the addition of buffer ATL and proteinase K, the 56 °C incubation time was increased to ~16 h, i.e. samples were incubated overnight; the volume of elution buffer AE was decreased to 50 μl to increase the gDNA concentration; and the elution step was repeated on the 50 μl eluate to increase the gDNA yield. Extraction reagent blank controls (ExCs) (*n* = 21) were included alongside each batch of gDNA extractions.

### PCR amplification

#### Initial piroplasm and *Hepatozoon* spp. screening

The 711 gDNA samples from ticks and 21 ExCs were initially screened for a short 300 bp region of the *18S* ribosomal RNA gene (*18S*) of piroplasms and *Hepatozoon* spp. with the primers 18SApiF/18SApiR (Table 2) that were designed in Geneious v10.2.2 [10] based on an alignment of piroplasms and *Hepatozoon* species. Conventional PCRs were performed in 25 μl reaction volumes with 1× KAPA Taq buffer (Sigma-Aldrich, St. Louis, Missouri, USA), 2 mM MgCl_2_, 1 mM dNTPs, 0.04 mg BSA (Fisher Biotec, Perth, Western Australia, Australia), 400 nM of each forward and reverse primer, 0.02 U KAPA Taq DNA Polymerase (Sigma-Aldrich) and 1 μl of neat gDNA. Thermal cycling conditions included an initial denaturation at 95 °C for 5 min followed by 40 cycles of denaturation at 95 °C for 30 s, annealing at 58 °C for 30 s and extension at 72 °C for 45 s, with a final extension of 72 °C for 5 min. No-template controls (NTCs) were included alongside all PCR assays.

**Table 2:** Summary of primer properties

Target gene	Target organisms	Primer name	Primer sequence (5'-3')	Expected amplicon length (bp)	Annealing temperature (°C)	Reference
*18S*	Piroplasms and *Hepatozoon* spp.	18SApiF	ACGAACGAGACCTTAACCTGCTA	300	58	This study
		18SApiR	GGATCACTCGATCGGTAGGAG			
	*Babesia* spp. and *Theileria* spp.	BTF1 (external)	GGCTCATTACAACAGTTATAG	930	58	[11]
		BTR1 (external)	CCCAAAGACTTTGATTTCTCTC			
		BTF2 (internal)	CCGTGCTAATTGTAGGGCTAATAC	800	62	
		BTR2 (internal)	GGACTACGACGGTATCTGATCG			
		Nbab_1F	AAGCCATGCATGTCTAAGTATAAGCTTTT	1500	60	[13]
		18SApiR	GGATCACTCGATCGGTAGGAG			This study
	*Hepatozoon* spp.	HepF300	GTTTCTGACCTATCAGCTTTCGACG	600	60	[16]
		Hep900	CAAATCTAAGAATTTCACCTCTGAC			
		HEMO1	TATTGGTTTTAAGAACTAATTTTATGATTG	900	48	[17]
		HEMO2	CTTCTCCTTCCTTTAAGTGATAAGGTTCAC			
		HAM-1F	GCCAGTAGTCATATGCTTGTC	1650	56	[18]
		HPF-2R	GACTTCTCCTTCGTCTAAG			
MPSP p32	*Theileria orientalis*	Ts-U	CACGCTATGTTGTCCAAGAG	800	58C	[15, 19]^a^
		Ts-R	TGTGAGACTCAATGCGCCTA			

^a^Primers were designed by Tanaka et al. [19] and PCR assay conditions were followed according to Zakimi et al. [15]

#### *Babesia* spp. and *Theileria* spp. amplification

All samples that were 18SApiF/18SApiR-positive were sequenced using Sanger sequencing and apicomplexan species were identified using the Basic Local Alignment Search Tool (BLAST) for sequence comparison to the National Centre for Biotechnology Information (NCBI) non-redundant nucleotide (nr/nt) database (methods described in later sections). Samples that were positive for *B. vogeli*, *Hepatozoon* spp. or had mixed sequence chromatograms from the 18SApiF/18SApiR PCR screening were then subjected to a nested *Babesia* spp. and *Theileria* spp. PCR assay using the external primers BTF1/BTR1 that target a 930 bp region of *18S* and the internal primers BTF2/BTR2 that target an 800 bp region of *18S* [11] (Table 2). The PCR assays were carried out according to the 18SApiF/18SApiR PCR assay conditions described in the present study, with the following modification: the final MgCl_2_ concentration was 1.5 mM. The thermal cycling conditions for the BTF1/BTR1 and BTF2/BTR2 primer sets were carried according to previously described methodologies [11], with the following modification: the denaturation temperature was increased to 95 °C for BTF1/BTR1 and BTF2/BTR2.

Following unsuccessful attempts to achieve amplification of a long (> 1300 bp) region of *18S* for all samples that were positive for novel *Babesia* and *Theileria* species with the previously published primer sets BT18SF1/BT18SR1 and BT18SF2/BT18SR2 (nested PCR) [12], and Nbab_1F [13] and TB-Rev [14], different primer combinations were tested that had similar melting temperatures (≤ 5 °C). The primer combinations included BTF1/TB-Rev [11, 15], BTF1/BT18SR2 [11, 12] and Nbab_1F/18SApiR [12, 13]. PCR assays were carried out according to the methods described for 18SApiF/18SApiR, with the following modifications: reaction volumes were increased to 50 μl and 2 μl of *Theileria orientalis* Yakimov and Sudachenkov, 1931 genotype Ikeda positive control gDNA was used. Thermal cycling conditions included an initial denaturation at 95 °C for 5 min followed by 50 cycles of denaturation at 95 °C for 30 s, annealing temperature (Tann) gradients were carried out ranging from 48–60 °C for 30 s and extension at 72 °C for 2 min, with a final extension of 72 °C for 5 min. Amplification of a ~1500 bp product was observed in the *T. orientalis* genotype Ikeda positive control for the Nbab_1F/18SApiR primers with a Tann of 60 °C (Table 2). These methods, with the Nbab_1F/18SApiR primers and Tann of 60 °C, were then used to amplify samples positive for novel *Babesia* and *Theileria* species using conventional PCR.

#### *Hepatozoon* spp. amplification

Samples that were positive for piroplasms, *Hepatozoon canis* James, 1905 and those that had mixed sequence chromatograms from the 18SApiF/18SApiR screening were then subjected to *Hepatozoon* spp. PCR assays using two different primer sets; HepF300/Hep900 [16] and HEMO1/HEMO2 [17] that target a 600 bp and 900 bp region of *18S*, respectively (Table 2). The PCR assays for the HepF300/Hep900 primers were carried out according to the methods described for the 18SApiF/18SApiR primers in the present study, with the following modification: the final MgCl_2_ concentration was decreased to 1.5 mM. The thermal cycling conditions were followed according to previously published methods [16], with the following modifications: the denaturation temperature was increased to 95 °C; the number of cycles was increased to 40; and the final extension time was decreased to 5 min. For the HEMO1/HEMO2 primer set, the PCR assays and thermal cycling conditions were carried out using the 18SApiF/18SApiR methods described in the present study, with the following modifications: the number of cycles was increased to 45; the optimal Tann was determined by a Tann gradient to be 48 °C; and a 1 min extension time was used during the 45 cycles.

A ~1650 bp *18S* region of the novel *Hepatozoon* species was amplified using the HAM-1F/HPF-2R primer set [18] (Table 2). The PCR assay and thermal cycling conditions described above for the Nbab_1F/18SApiR primers were used, but with a Tann of 56 °C.

#### *Theileria* orientalis genotyping

To determine the genotypes of *T. orientalis*, the Ts-U/Ts-R primers [19] were used to amplify 800 bp of the major piroplasm surface protein (MPSP) gene of *T. orientalis* that encodes MPSP p32 (Table 2). The PCR assays were carried out according to the 18SApiF/18SApiR methods described in this study. The thermal cycling conditions were followed according to previously published methods [15], with the following modifications: the denaturation temperature was increased to 95 °C; the number of cycles was increased to 40; and the final extension time was increased to 5 min.

### Gel electrophoresis and PCR product purification

The amplified DNA was electrophoresed in 1% agarose gel containing SYBR Safe Gel Stain (Invitrogen, Carlsbad, California, USA) and visualized with a dark reader trans-illuminator (Clare Chemical Research, Dolores, Colorado, USA). PCR products of the expected amplicon size were excised from the gel with sterile scalpel blades and purified for Sanger sequencing using a filtered pipette tip method [20].

### Sanger sequencing

Purified PCR products were sequenced in forward and reverse directions independently on a 96-capillary 3730xl DNA Analyzer (Thermo Fisher Scientific, Waltham, Massachusetts, USA) using an ABI Prism™ BigDye v3.1. Cycle Sequencing kit (Applied Biosystems, Foster City, California, USA) according to the manufacturer’s instructions.

### Phylogenetic analyses

Forward and reverse sequence chromatograms were aligned and merged to generate consensus sequences and were trimmed of primers using Geneious v10.2.2. BLAST was used to compare the consensus sequences to the NCBI nr/nt database. For phylogenetic analyses of piroplasm, *Hepatozoon* and the Coccidiasina: Eucoccidiorida, cf. Sarcocystidae sp. consensus sequences, the longest available *18S* sequences on GenBank for named Piroplasmida, Adeleorina and Sarcocystidae species were imported into Geneious v10.2.2 and aligned using the MUSCLE alignment tool [21]. As partial *18S* sequence lengths varied, the alignments were trimmed to retain as many named species as possible in overlapping hypervariable regions, but some sequences were removed from the alignment due to either their short length, or the region did not overlap with the majority of other sequences.

Phylogenetic analyses of piroplasm and *Hepatozoon* consensus sequences were carried out that also included GenBank sequences with ≥ 95% and ≥ 96% similarity, respectively, over a greater nucleotide alignment length. After sequences in these alignments were trimmed to the length of the shortest sequence with ≥ 95% or ≥ 96% similarity, duplicate sequences were removed. An alignment of Eucoccidiorida *18S* sequences was generated to phylogenetically assess the grouping of the consensus cf. Sarcocystidae sp. sequence respective to other families of the Eucoccidiorida, and this alignment was trimmed to the length of the consensus cf. Sarcocystidae sp. sequence (572 bp).

Nucleotide alignments were imported into the program PhyML [22] and assessed for the most appropriate nucleotide substitution model based on Bayesian Information Criterion (BIC) and Bayesian phylogenetic trees were constructed using MrBayes v3.2.6 [23].

Sequences generated from this study have been submitted to GenBank under the accession numbers MG062865, MG571580-MG571582, MG593271-MG593276, MG758109-MG758121 and MG758124-MG758138.

### Genetic distance estimates

The longer *18S* fragment sequences from the novel species found in this study were compared to the NCBI nr/nt database using BLAST, and the *18S* sequences from the most closely related unnamed and named species were imported into Geneious v10.2.2 for genetic pairwise distance (percent sequence identity) comparisons. The *18S* sequences of the most closely related named species were then compared to the NCBI nr/nt database using BLAST, and the *18S* sequences with the highest percent similarity to the named species were also imported into Geneious v10.2.2 for pairwise distance comparisons. As partial *18S* sequences were obtained in the present study, the effect of shorter versus longer *18S* alignments on pairwise distance estimates was assessed by comparing the percentage differences in pairwise identities between shorter (~1500 bp) and longer (~1650 bp) alignments of the most closely related named species. The program MUSCLE was used to build the alignments and the pairwise percent identities were calculated with a Kimura distance matrix [21].

## Results

### Prevalence of Apicomplexa species

Approximately 300 bp of *18S* was amplified in 41/711 samples using the 18SApiF/18SApiR primer set; however, only 16/41 of these 18SApiF/18SApiR-positive samples had good quality chromatograms, while the rest had mixed chromatograms as a result of amplification of multiple eukaryotic organisms. No amplification was observed in the ExCs or NTCs for any of the PCR assays. The sequence accession numbers, lengths and top BLAST matches for all sequences obtained in this study are summarised in Additional file 2: Table S2.

Of the 26 samples for which mixed chromatograms were obtained using the 18SApiF/18SApiR primer set, *Hepatozoon* spp. PCRs yielded no amplification. Amplification with the BTF1/BTR1 and BTF2/BTR2 primer set identified one positive from the 26 samples (*Haemaphysalis longicornis* Neumann nymph, sample HLN3) that had mixed chromatograms. Additionally, mixed infections with *Hepatozoon* and *Theileria* species were detected in two *Ixodes tasmani* Neumann samples (*Hepatozoon banethi* n. sp. and *Theileria apogeana* n. sp. in sample ITF7; *H. banethi* n. sp. and *Theileria palmeri* n. sp. in sample ITF6) at an overall prevalence of 0.3% (2/711; 95% CI: 0–1.0%). A mixed infection with *Theileria* species (*T. palmeri* n. sp. and *Theileria paparinii* n. sp.) was detected in one *I. tasmani* sample (ITF1) (0.1%; 1/711; 95% CI: 0–0.8%). The overall prevalence of the Apicomplexa species in different states and territories, and Australia-wide, in all tick species from all host species is summarised in Table 3. The prevalence of Apicomplexa species Australia-wide and in each state and territory is summarised for all tick species from all hosts, individual tick species from all hosts, and individual tick species from dogs, cats and horses in Additional file 3: Table S3.

**Table 3:** Prevalence of Apicomplexa species in different states and territories, and Australia-wide, in all tick species from all host species

Apicomplexa species	Number of positives/sample size (percent proportion, 95% confidence interval^a^)							
	NSW	NT	QLD	SA	TAS	VIC	WA	Australia
*Babesia lohae* n. sp.	–	–	1^b^/171 (0.6; 0–3.2)	–	–	–	–	1/711 (0.1; 0–0.8)
*Babesia mackerrasorum* n. sp.	1^c^/293 (0.3; 0-1.9)	–	-	–	–	–	–	1/711 (0.1; 0–0.8)
*Babesia canis vogeli*	–	1^d^/50 (2.0; 0.1–10.6)	1^e^/171 (0.6; 0–3.2)	–	–	–	–	2/711 (0.3; 0–1)
*Hepatozoon canis*	–	–	1^f^/171 (0.6; 0–3.2)	–	–	–	–	1/711 (0.1; 0–0.8)
*Hepatozoon banethi* n. sp.	–	–	–	–	3^g^/65 (4.6; 1–12.9)	–	–	3/711 (0.4; 0.1–1.2)
*Hepatozoon ewingi* n. sp.	1^h^/293 (0.3; 0–1.9)	–	–	–	-	–	–	1/711 (0.1; 0–0.8)
*Theileria apogeana* n. sp.	–	–	–	–	1^i^/65 (1.5; 0–8.3)	–	–	1/711 (0.1; 0–0.8)
*Theileria orientalis* genotype Ikeda	3^j^/293 (1.0; 0.2–3)	–	–	–	-	–	–	3/711 (0.4; 0.1–1.2)
*Theileria palmeri* n. sp.	–	–	–	–	2^k^/65 (3.1; 0.4–10.7)	–	–	2/711 (0.3; 0–1)
*Theileria paparinii* n. sp.	–	–	–	–	2^l^/65 (3.1; 0.4–10.7)	–	–	2/711 (0.3; 0–1)
*Theileria worthingtonorum* n. sp.	–	–	–	–	2^m^/65 (3.1; 0.4–10.7)	–	–	2/711 (0.3; 0–1)
Coccidiasina: Eucoccidiorida, cf. Sarcocystidae sp.	1^n^/293 (0.3; 0–1.9)	–	–	–	–	–	–	1/711 (0.1; 0–0.8)
Mixed infections with *Theileria* and *Hepatozoon* species	–	–	–	–	2^o^/65 (3.1; 0.4–10.5)	–	–	2/711 (0.3; 0–1)
Mixed infections with *Theileria* species	–	–	–	–	1^p^/65 (1.5; 0–8.3)	–	–	1/711 (0.1; 0–0.8)
Total Apicomplexa species prevalence	6/293 (2.0; 0.8–4.4)	1/50 (2.0; 0.1–10.6)	3/171 (1.8; 0.4–5.0)	0/48 (0; 0–7.4)	10/65 (15.4; 7.6–26.5)	0/11 (0; 0–28.5)	0/73 (0; 0–4.9)	20/711 (2.8; 1.7–4.3)

^a^95% confidence intervals were calculated based on the methods by Rozsa et al. [48]

^b^Detected in *Ixodes holocyclus* from a cat (*n* = 1)

^c^Detected in cf. *Haemaphysalis* sp. from a horse (*n* = 1)

^d^Detected in *Rhipicephalus sanguineus* from a dog (*n* = 1)

^e^Detected in *Rhipicephalus sanguineus* from a dog (*n* = 1)

^f^Detected in *Ixodes holocyclus* from a dog (*n* = 1)

^g^Detected in *Ixodes tasmani* from dogs (*n* = 3)

^h^Detected in *Haemaphysalis bancrofti* from a horse (*n* = 1)

^i^Detected in *Ixodes tasmani* from dogs (*n* = 2)

^j^Detected in *Haemaphysalis longicornis* from dogs (*n* = 3)

^k^Detected in *Ixodes tasmani* from dogs (*n* = 2)

^l^Detected in *Ixodes tasmani* from dogs (*n* = 2)

^m^Detected in *Ixodes tasmani* from dogs (*n* = 2)

^n^Detected in *Ixodes tasmani* from a dog (*n* = 1)

^o^Detected in *Ixodes tasmani* from dogs (*n* = 2)

^p^Detected in *Ixodes tasmani* from a dog (*n* = 1)

Overall, of the 41 samples that were positive by PCR, sequences were unambiguously confirmed in 17/711 samples (2.4%; 95% CI: 1.4–3.8%). The overall prevalence of the 12 Apicomplexa species (including the three mixed infections) based on confirmed sequences was 2.8% (20/711; 95% CI: 1.4–3.8%) (Table 3).

### Endemic tick-borne pathogens: *B. vogeli* and *T. orientalis*

Two known endemic tick-borne pathogens were identified: *B. vogeli* and *T. orientalis* genotype Ikeda. *Babesia vogeli* (~300 bp) (100% homology) was detected in 1.1% of *R. sanguineus* ticks collected from dogs (2/184; 95% CI , 0.1–3.9%) (Additional file 3: Table S3); an *R. sanguineus* female from a dog in Queensland (QLD) (sample RSF1; MG758129) (3%; 1/33); 95% CI: 0.1–15.8%), and an *R. sanguineus* larva from a dog in the Northern Territory (NT) (sample RSL1; MG758131) (2%; 1/50; 95% CI: 0.1–10.6%) (Additional file 3: Table S3). Further characterisation of longer *18S* sequences obtained from RSF1 (MG758130) and RSL1 (MG758132) with the nested piroplasm PCR assay confirmed their 100% homology to *B. vogeli* isolates in GenBank (Additional file 2: Table S2).

*Theileria orientalis* genotype Ikeda (100% homology) was detected in three *H. longicornis* nymphs at the *18S* locus (HLN1-3) (MG571580-MG571582) at a prevalence of 4% (3/75; 95% CI: 0.8–11.2%) (Additional file 3: Table S3). These *Theileria orientalis* genotype Ikeda-positive *H. longicornis* nymphs were removed from dogs in New South Wales (NSW) (6.4%; 3/47; 95% CI: 1.3–17.5%) (Additional file 3: Table S3). Amplification of these samples at the MPSP p32 gene locus produced 835 bp sequences (MG758109-MG758111), which were 100% identical to each other, and 100% identical to *Theileria orientalis* strain Shintoku, genotype Ikeda (XM_009691550) (Additional file 2: Table S2).

### An exotic tick-borne pathogen: *H. canis*

Unexpectedly, a 303 bp sequence with 100% sequence similarity to the exotic tick-borne pathogen, *H. canis*, was obtained from one engorged *Ixodes holocyclus* Neumann female (sample IHF2; MG062865) (0.3%; 1/335; 95% CI: 0–1.7%) removed from a dog in QLD (1.7%; 1/60; 95% CI: 0–8.9%). Further characterisation of this sample using the HepF300/Hep900 and HEMO1/HEMO2 primer sets produced sequences with a ~30 bp overlap, and this concatenated sequence (MG758124) was 1409 bp in length and was 99.9% similar, with two single nucleotide polymorphisms (SNPs), to *H. canis* (KX712124) (Additional file 2: Table S2).

### Novel *Babesia* species

A novel *Babesia* species, *Babesia mackerrasorum* n. sp., was identified in a cf. *Haemaphysalis* Koch sp. male from a horse in NSW (sample HspM1; MG593271 and MG593276) (33.3%; 1/3; 95% CI: 0.8–90.6%) with 98.3% similarity to *Babesia macropus* Dawood, 2013 (JQ437265) isolated from an eastern grey kangaroo (*Macropus giganteus* Shaw) in NSW, Australia. Another novel *Babesia* species, *Babesia lohae* n. sp., was identified in an *I. holocyclus* female from a cat in QLD (sample IHF1; MG593272 and MG593273) (2%; 1/51; 95% CI: 0–10.4%), which was only 96.7% similar to *B. mackerrasorum* n. sp. (MG593271) (refer to pairwise genetic distance matrix in Additional file 4: Table S4), and 100% similar to *Babesia* sp. (MG251436) isolated from *I. tasmani* collected from a brushtail possum (*Trichosurus vulpecula* Kerr) in Australia (Additional file 2: Table S2).

### Novel *Hepatozoon* species

Three *I. tasmani* ticks removed from dogs in TAS contained a novel *Hepatozoon* species; *H. banethi* n. sp. [samples ITF2 (MG758133 and MG758134), ITF6 (MG758135 and MG758136) and ITF7 (MG758138 and MG758137)] (6.8%; 3/44; 95% CI: 1.4–18.7%) (Additional file 3: Table S3). The long *18S H. banethi* n. sp. sequences from ITF6 (MG758136) and ITF7 (MG758137) were 99.9% similar to each other, and the long *18S H. banethi* n. sp. sequence from ITF2 (MG758133) was 99.8% similar to the sequences from ITF6 and ITF7 (Additional file 4: Table S4). The top NCBI BLAST results revealed that the long *18S* sequences of *H. banethi* n. sp. were most similar (98.0% for ITF2 and ITF6, and 97.8% for ITF7) to *Hepatozoon* sp. (FJ719813) isolated from a colocolo opossum (*Dromiciops gliroides* Thomas) in Chile (Additional file 2: Table S2). *Hepatozoon ewingi* n. sp. was detected in *Haemaphysalis bancrofti* Nuttall and Warburton from a horse in NSW (sample HBM1; MG593274 and MG593275) (33.3%; 1/3; 95% CI: 0.8–90.6%) (Additional file 3: Table S3). The long *18S* sequence of *H. ewingi* n. sp. (MG593275) was only 94.8% similar to *H. banethi* n. sp. sequences from ITF2 (MG758133) and ITF6 (MG758136) and 94.5% similar to the *H. banethi* n. sp. sequence from ITF7 (MG758137) (Additional file 4: Table S4), and was also most similar (96.3%) to *Hepatozoon* sp. (FJ719813) isolated from *D. gliroides* (Additional file 2: Table S2).

### Novel *Theileria* species

Four novel *Theileria* species were found in *I. tasmani* ticks from dogs in Tasmania (TAS): *T. apogeana* n. sp. from sample ITF7 (MG758116 and MG758126) (2.3; 1/44; 95% CI: 0.1–12.0%); *T. palmeri* n. sp. from samples ITF1 (MG758113) and ITF6 (MG758120 and MG758125) (4.5%; 2/44; 95% CI: 0.6–15.5%); *T. paparinii* n. sp. from samples ITF1 (MG758112) and ITF4 (MG758115 and MG758117) (4.5%; 2/44; 95% CI, 0.6–15.5%); and *Theileria worthingtonorum* n. sp. from samples ITF3 (MG758114 and MG758118) and ITF5 (MG758119 and MG758121) (4.5%; 2/44; 95% CI: 0.6–15.5%). The interspecific genetic distances of the long *18S* sequences ranged from 2.8% between *T. apogeana* n. sp. and *T. paparinii* n. sp. to 6.9% between *T. apogeana* n. sp. and *T. palmeri* n. sp. (Additional file 4: Table S4). The long *18S* sequence of *T. apogeana* n. sp. (MG758116) was most similar (96.5%) to *Theileria* sp. (JQ682879) isolated from a burrowing bettong (*Bettongia lesueur* Quoy and Gaimard) in Western Australia (WA), and *T. paparinii* n. sp. (MG758115) was most similar (98.9%) to the same *Theileria* sp. isolate from *B. lesueur* (JQ682879) (Additional file 2: Table S2). *Theileria palmeri* n. sp. (MG758113 and MG758120) was most similar (95.8%) to *Theileria* sp. (MF576261) isolated from *Ixodes australiensis* Neumann in Australia, and *T. worthingtonorum* n. sp. (MG758114 and MG758121) was most similar (98.3%) to the same *Theileria* sp. isolate from *I. australiensis* (MF576261) (Additional file 2: Table S2).

The long *18S* sequences for the novel piroplasms (~1450 bp) and *Hepatozoon* species (~1650 bp), as well as the short *18S* sequences obtained for novel species, are summarised in Table 4.

**Table 4:** Novel piroplasms and *Hepatozoon* species summary

Apicomplexan species	Tick species	Sample name	Host species	Type locality	GenBank ID
*Babesia lohae* n. sp.	*Ixodes holocyclus*	IHF1	Cat	Park Ridge, QLD	MG593272, MG593273
*Babesia mackerrasorum* n. sp.	cf. *Haemaphysalis*	HspM1	Horse	Tanja, NSW	MG593271, MG593276
*Hepatozoon banethi* n. sp.	*Ixodes tasmani*	ITF2	Dogs	Devonport, TAS	MG758133, MG758134
		ITF6^a^		Port Sorell, TAS	MG758135, MG758136
		ITF7^b^		Devonport, TAS	MG758137, MG758138
*Hepatozoon ewingi* n. sp.	*Haemaphysalis bancrofti*	HBM1	Horse	Eungai Creek, NSW	MG593274, MG593275
*Theileria apogeana* n. sp.	*Ixodes tasmani*	ITF7^b^	Dog	Devonport, TAS	MG758116, MG758126
*Theileria palmeri* n. sp.	*Ixodes tasmani*	ITF1^c^	Dogs	Devonport, TAS	MG758113
		ITF6^a^		Port Sorell, TAS	MG758120, MG758125
*Theileria paparinii* n. sp.	*Ixodes tasmani*	ITF1^c^	Dogs	Devonport, TAS	MG758112
		ITF4		Lower Wilmot, TAS	MG758115, MG758117
*Theileria worthingtonorum* n. sp.	*Ixodes tasmani*	ITF3	Dogs	Port Sorell, TAS	MG758114, MG758118
		ITF5		Lower Wilmot, TAS	MG758119, MG758121

^a^Co-infection with *H. banethi* n. sp. and *T. palmeri* n. sp.

^b^Co-infection with *H. banethi* n. sp. and *T. apogeana* n. sp.

^c^Co-infection with *T. palmeri* n. sp. and *T. paparinii* n. sp.

### Novel cf. Sarcocystidae gen. sp.

The short *18S* cf. Sarcocystidae Poche, 1913 gen. sp. sequence detected in an *I. tasmani* tick from a dog in NSW (sample ITF8; MG758127) (100%; 1/1; 95% CI, 2.5–100%) was most similar (98.4%) to species from several different genera in the family Sarcocystidae, including *Besnoitia darlingi* Mandour, 1965 (MF872603), *Toxoplasma gondii* Nicolle and Manceaux, 1908 (XR_001974356), *Hammondia heydorni* Dubey, 1977 (KT184370) and Eimeriidae Minchin, 1903 sp. (KJ634019). Amplification was not obtained for the cf. Sarcocystidae gen. sp. positive sample with the nested PCR, or the HEMO1/HEMO2 PCR, but a 572 bp product was amplified with the HepF300/Hep900 primers. Unexpectedly, a 572 bp cf. Sarcocystidae gen. sp. sequence was obtained (MG758128) and was most similar (96.5%) to *Besnoitia* Henry, 1913 spp. in GenBank; the top BLAST match was for *Besnoitia besnoiti* Henry, 1913 (KJ746531) isolated from cattle (*Bos taurus* Linnaeus) in Croatia (Additional file 2: Table S2).

### Genetic distance estimates

The pairwise percent identities of the novel species to the most closely related unnamed species in GenBank ranged from 95.8% between *T. palmeri* n. sp. (MG758112) and *Theileria* sp. (MF576261) to 100% between *B. lohae* n. sp. (MG593272) and *Babesia* sp. (MG251436) (Table 5). The pairwise identities between the novel and described species ranged from 92.1% between *T. palmeri* n. sp. (MG758112) and *Theileria bicornis* Nijhof*,* 2003 (AF499604) to 98.3% between *B. mackerrasorum* n. sp. (MG593271) and *B. macropus* (JQ437265). The average pairwise identities for the novel *Babesia*, *Hepatozoon* and *Theileria* species to *18S* sequences from named species in GenBank were 97.4% (standard deviation (SD) 1.3%), 96.1% (SD 0.62%), and 92.5% (SD 0.5%), respectively. The pairwise identities were higher between the two most closely related named species compared to the pairwise identities between the novel species and most closely related named species in all cases, except for *B. mackerrasorum* n. sp.; the average was 98.3% (SD 1.35%), 96.9% (SD 0.02%) and 94.5% (SD 1.80%) for *Babesia*, *Hepatozoon* and *Theileria* species, respectively. The average pairwise identities for the longer alignments were 98.5% (SD 0.85%), 97.0% (SD 0%) and 94.4% (SD 1.93%) for *Babesia*, *Hepatozoon* and *Theileria* species, respectively. The percentage differences between the pairwise similarities of the shorter *versus* longer alignments were low, with pairwise identities being overestimated by a maximum of 0.3% and underestimated by a maximum of 0.6% in the shorter alignment (Table 5).

**Table 5:** Pairwise genetic identities of long *18S* rRNA gene sequences from this study compared to the most closely related unnamed and named species in GenBank

Species name (Accession number)	Sequence length (bp)	Most similar unnamed species		Most similar named species		Closest relative to described species (Accession number)	Pairwise identity between the most closely related named species				
							Partial *18S* alignment trimmed to this study’s sequence length		Near full-length *18S* alignment		Percentage differences (%)^c^
		Sequence name (Accession number)	Pairwise identity to this study’s sequence (%)	Sequence name (Accession number)	Pairwise identity to this study’s sequence (%)		Alignment length (bp)	Pairwise identity (%)	Alignment length (bp)	Pairwise identity (%)	
*Babesia lohae* n. sp. (MG593272)	1430	*Babesia* sp. isolate BP7 (MG251436)	100	*Babesia orientalis* strain DaYe (HQ840969)	96.4	*Babesia occultans* isolate 58 (HQ331478)	1430	99.2	1577	99.1	-0.1
*Babesia mackerrasorum* n. sp. (MG593271)	1431	*Babesia* sp. Kashi 2 (AY726557)	96.5	*Babesia* sp. ALT-2012 strain 3 NSW (JQ437265)^a^	98.3	*Babesia orientalis* strain DaYe (HQ840969)	1430	97.3	1634	97.9	0.6
*Hepatozoon banethi* n. sp. (MG758137)^b^	1679	*Hepatozoon* sp. DG1 (FJ719813)	97.8	*Hemolivia stellata* (KP881349)	96.6	*Hepatozoon ayorgbor* (EF157822)	1681	96.9	1775	97.0	0.1
*Hepatozoon ewingi* n. sp. (MG593275)	1680	*Hepatozoon* sp. DG1 (FJ719813)	96.3	*Hemolivia stellata* (KP881349)	95.7	*Hepatozoon ayorgbor* (EF157822)	1666	96.9	1775	97.0	0.1
*Theileria apogeana* n. sp. (MG758116)	1480	*Theileria* sp. K1 (JQ682879)	96.5	*Theileria bicornis* (AF499604)	92.2	*Cytauxzoon felis* (AF399930)	1498	93.7	1676	93.4	-0.3
*Theileria palmeri* n. sp. (MG758112)^b^	1506	*Theileria* sp. (MF576261)	95.8	*Theileria bicornis* (AF499604)	92.1	*Cytauxzoon felis* (AF399930)	1509	93.6	1676	93.4	-0.2
*Theileria paparinii* n. sp. (MG758115)	1496	*Theileria* sp. K1 (JQ682879)	98.9	*Theileria uilenbergi* isolate Li 2 (JF719835)	92.3	*Theileria cervi* isolate Wisconsin elk 3 clone 16 (AY735129)^d^	1480	97.2	1664	97.3	0.1
*Theileria worthingtonorum* n. sp. (MG758114)^b^	1504	*Theileria* sp. (MF576261)	98.3	*Theileria bicornis* (AF499604)	93.3	*Cytauxzoon felis* (AF399930)	1512	93.7	1676	93.4	-0.3

^a^ *Babesia macropus*

^b^The longest *18S* sequence was selected for the pairwise genetic distance analyses

^c^Percentage differences between pairwise identities of trimmed and near full-length *18S* alignment

^d^Complete *18S* sequence

### Phylogenetic analyses

**Fig. 1:**
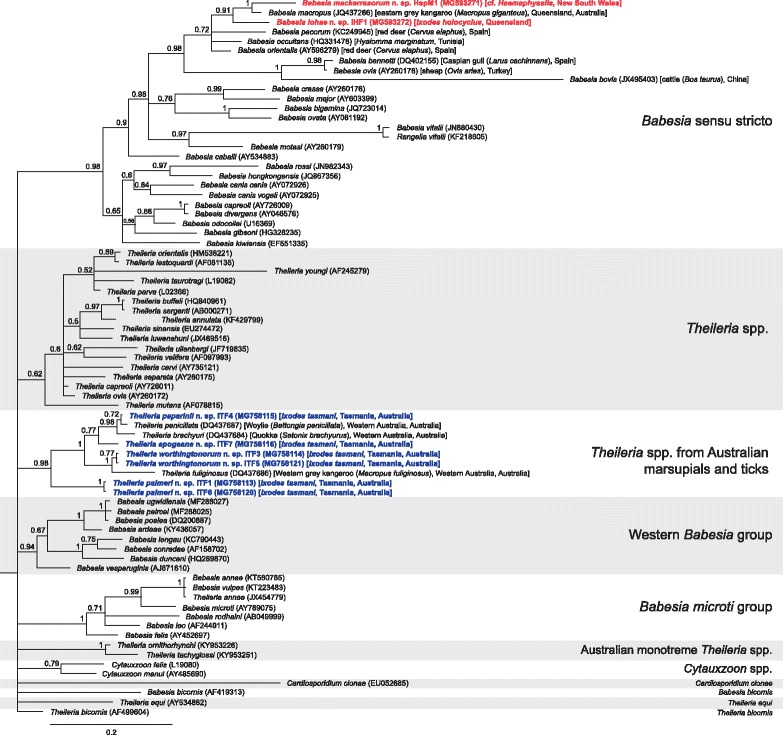
Bayesian phylogenetic tree of a 574 bp alignment of *18S* sequences of named piroplasm species and novel piroplasm sequences derived from this study. The tree was built using the following parameters: HKY85+G+I model; 1,100,000 Markov chain Monte Carlo (MCMC) length; ‘burn-in’ length of 10,000; subsampling frequency of 200. The tree was rooted with the outgroup sequence *Plasmodium falciparum* (JQ627152) (not shown). Scale-bar indicates the number of nucleotide substitutions per site

**Fig. 2:**
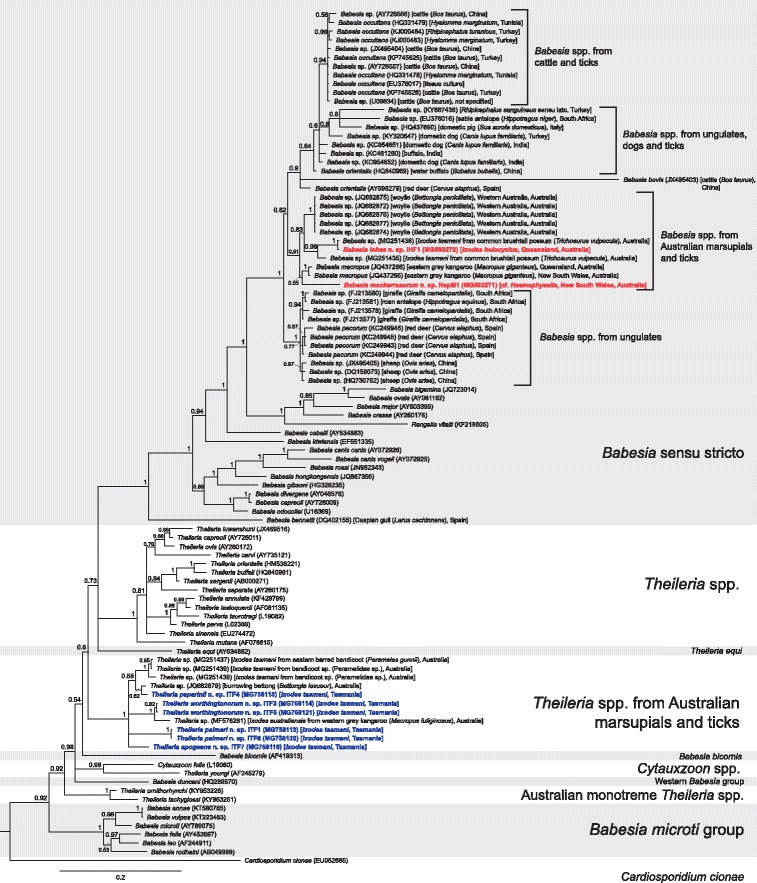
Bayesian phylogenetic tree of a 1720 bp alignment of *18S* sequences of named piroplasm species and novel piroplasm sequences derived from this study, with unnamed sequences with ≥ 95% similarity to novel species included. The tree was built using the following parameters: GTR+G+I model; 1,100,000 MCMC length; ‘burn-in’ length of 10,000; subsampling frequency of 200. The tree was rooted with the outgroup sequence *Plasmodium falciparum* (JQ627152) (not shown). Scale-bar indicates the number of nucleotide substitutions per site

The piroplasm phylogenetic tree of the novel species from the present study and named piroplasm species (Fig. 1) shows that *T. apogeana* n. sp., *T. palmeri* n. sp., *T. paparinii* n. sp. and *T. worthingtonorum* n. sp. grouped within a clade of *Theileria* species isolated from Australian marsupials with high support (posterior probabilities (pp) ≥ 0.72). *Babesia lohae* n. sp. and *B. mackerrasorum* n. sp. both grouped within the *Babesia sensu stricto* (*s.s.*) group and formed a clade with *B. macropus* (Fig. 1). When unnamed species with ≥ 95% were included in the phylogenetic tree and the length of the alignment was increased from 574 bp to 1720 bp to improve taxonomic resolution (Fig. 2), the novel *Theileria* species still grouped within a clade of sequences isolated from marsupials, but the clade became monophyletic with high support (pp = 1). Within this marsupial clade, *T. worthingtonorum* n. sp. formed a clade with *Theileria* sp. (MF576261) isolated from *I. australiensis* and *T. paparinii* n. sp. formed a clade with *Theileria* sp. (JQ682879) isolated from *B. lesueur*, while *T. palmeri* n. sp. and *T. apogeana* n. sp. did not group with any other sequences in the marsupial clade. *Babesia lohae* n. sp. grouped with other *Babesia* sp. sequences isolated from *I. tasmani* (MG251435 and MG251436) (pp ≥ 0.99), while *B. mackerrasorum* n. sp. grouped closest to, but distinct from, *B. macropus* isolates from *M. giganteus* in NSW and QLD (JQ437265 and JQ437266) (pp ≥ 0.55) (Fig. 2).

**Fig. 3:**
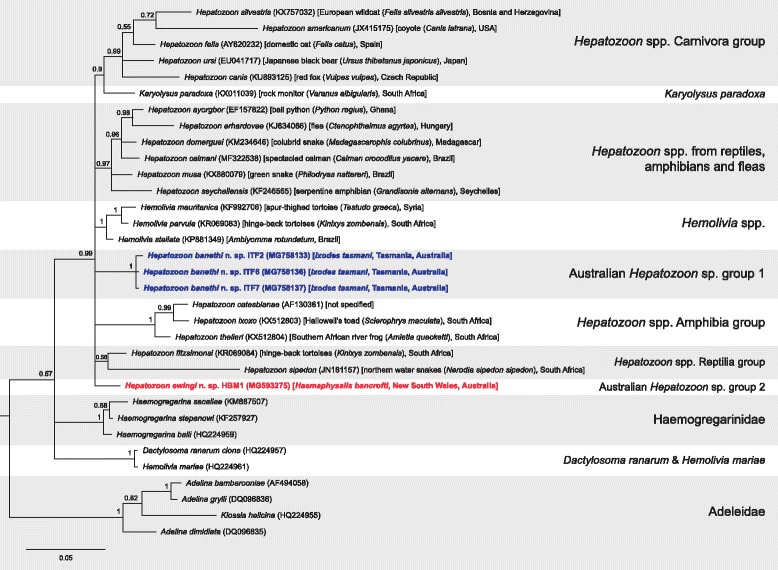
Bayesian phylogenetic tree of a 406 bp alignment of *18S* sequences of named Adeleorina species and novel *Hepatozoon* sequences derived from this study. The tree was built using the following parameters: GTR+G+I model; 1,100,000 MCMC length; ‘burn-in’ length of 10,000; subsampling frequency of 200. The tree was rooted with the outgroup sequence *Cryptosporidium serpentis* (AF151376) (not shown). Scale-bar indicates the number of nucleotide substitutions per site

**Fig. 4:**
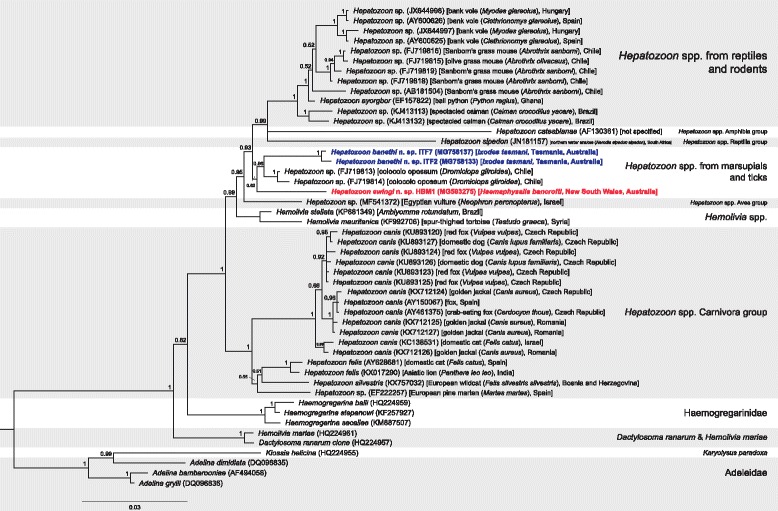
Bayesian phylogenetic tree of a 1457 bp alignment of *18S* sequences of named Adeleorina species and novel *Hepatozoon* sequences derived from this study, with unnamed sequences with ≥ 96% similarity to novel species included. The tree was built using the following parameters: GTR+G+I model; 1,100,000 MCMC length; ‘burn-in’ length of 10,000; subsampling frequency of 200. The tree was rooted with the outgroup sequence *Cryptosporidium serpentis* (AF151376) (not shown). Scale-bar indicates the number of nucleotide substitutions per site

The Adeleorina Léger, 1911 phylogenetic tree constructed from a 406 bp alignment that included novel *Hepatozoon* species from the present study and described Adeleorina species (Fig. 3) showed that *H. banethi* n. sp. and *H. ewingi* n. sp. grouped with other *Hepatozoon* species with high support (pp = 0.99) and also formed their own distinct clades. When closely related sequences (≥ 96% similar) were included in the phylogenetic reconstruction with a longer alignment length (1457 bp) (Fig. 4), *H. banethi* n. sp. and *H. ewingi* n. sp. formed a monophyletic clade with *Hepatozoon* sp. sequences isolated from *D. gliroides* (FJ719813 and FJ719814) (pp = 0.62).

**Fig. 5:**
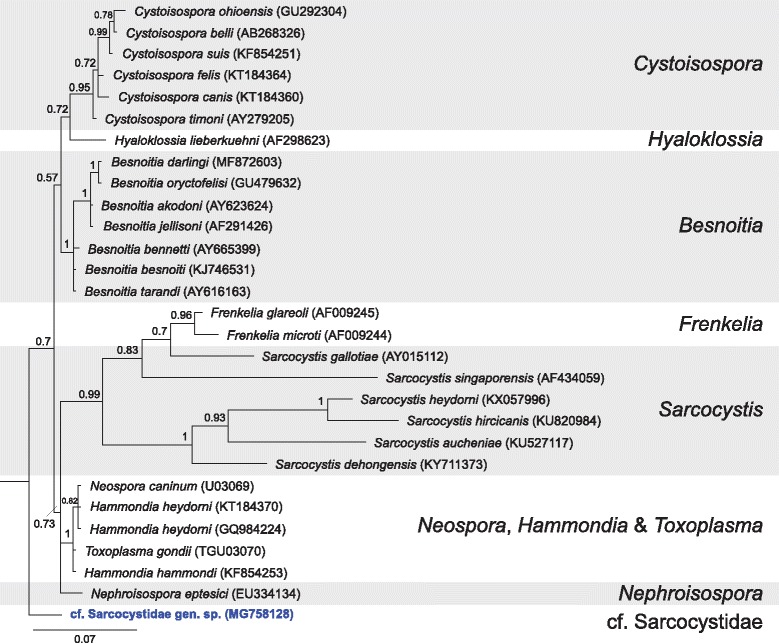
Bayesian phylogenetic tree of a 629 bp alignment of *18S* sequences of named Sarcocystidae species, with fewer *Sarcocystis* spp. included and the novel cf. Sarcocystidae sp. sequence derived from this study. The tree was built using the following parameters: GTR+G+I model; 1,100,000 MCMC length; ‘burn-in’ length of 10,000; subsampling frequency of 200. The tree was rooted with the outgroup sequence *Eimeria necatrix* (KT184349) (not shown). Scale-bar indicates the number of nucleotide substitutions per site

**Fig. 6:**
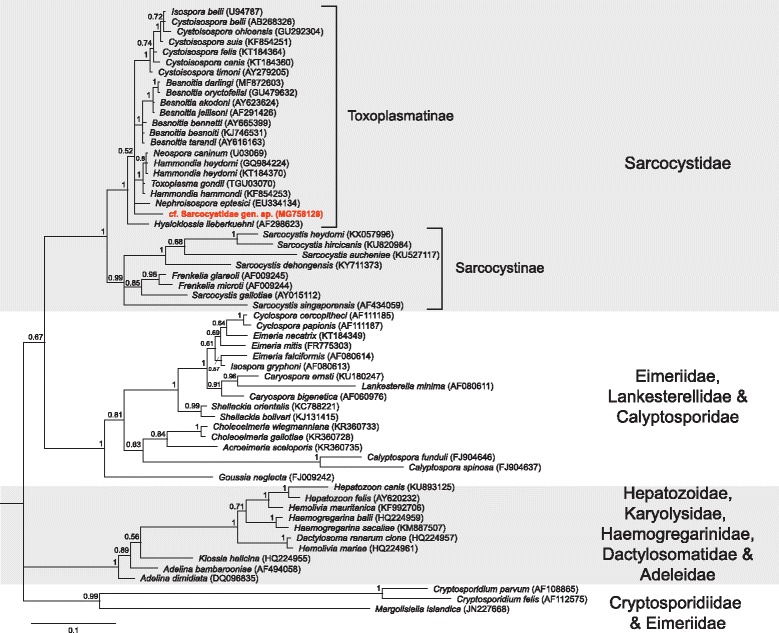
Bayesian phylogenetic tree of a 668 bp alignment of *18S* sequences of Eucoccidiorida families and the novel cf. Sarcocystidae sp. sequence. The tree was built using the following parameters: HKY85+G+I model; 1,100,000 MCMC length; ‘burn-in’ length of 10,000; subsampling frequency of 200. The tree was rooted with the outgroup sequence *Babesia rodhaini* (AB049999) (not shown). Scale-bar indicates the number of nucleotide substitutions per site

The phylogenetic tree of the cf. Sarcocystidae gen. sp. sequence from the present study and named Sarcocystidae species shows the cf. Sarcocystidae gen. sp. sequence was distinct to all other members of the Sarcocystidae family with low to moderate support (pp = 0.7) (Fig. 5). The phylogenetic tree of cf. Sarcocystidae gen. sp. and members of Eucoccidiorida families showed that cf. Sarcocystidae gen. sp. grouped with the Sarcocystidae family clade with Toxoplasmatinae spp. (pp = 0.52) (Fig. 6).

### Species descriptions

- **Order Piroplasmida Wenyon, 1926**
- **Suborder Piroplasmorina Levine, 1971**
- **Family Babesiidae Poche, 1913**
- **Genus** ***Babesia*** **Starcovici, 1893**

### *Babesia lohae* n. sp.

- ***Type-host*:** *Ixodes holocyclus* Neumann (Acari: Ixodidae).
- ***Type-locality*:** Park Ridge, Queensland, Australia.
- ***Type-material*:** Bisected tick, tissue extractions and genomic DNA were deposited at the Queensland Museum, Brisbane, Australia, under the accession numbers QMS108579, A015180 and A015181.
- ***Representative DNA sequences*:** DNA sequences were deposited in GenBank under the accessions MG593273 (299 bp *18S* rRNA gene) and MG593272 (1430 bp *18S* rRNA gene).
- ***Vector*:** The vector potential of *I. holocyclus* for *B. lohae* n. sp. is unknown.
- ***ZooBank registration*:** To comply with the regulations set out in article 8.5 of the amended 2012 version of the *International Code of Zoological Nomenclature* (ICZN) [24], details of the new species have been submitted to ZooBank. The Life Science Identifier (LSID) of the article is urn:lsid:zoobank.org:pub:D1B6E4E1-168C-488B-B809-381642900749. The LSID for the new name *Babesia lohae* n. sp. is urn:lsid:zoobank.org:act:7526D345-A3A5-4483-8AB6-599F76F0DA32.
- ***Etymology*:** This species is named for Ms Siew-May Loh who discovered *Babesia lohae* n. sp. in a separate study at the same time as the authors of the present study.

### Diagnosis

This organism is a species of *Babesia* (*s.s.*) that is genetically distinct from other described *Babesia* species and forms a clade with other *Babesia* (*s.s.*) species isolated from Australian marsupials and ticks (see above).

### *Babesia mackerrasorum* n. sp.

- ***Type-host*:** cf. *Haemaphysalis* sp. Koch (Acari: Ixodidae).
- ***Type-locality*:** Tanja, New South Wales, Australia.
- ***Type-material:*** Tissue extractions and genomic DNA were deposited at the Australian Museum, Sydney, Australia, under the accession numbers KS.128103.001 and KS.128103.002.
- ***Representative DNA sequences*:** DNA sequences were deposited in GenBank under the accessions MG593276 (299 bp *18S* rRNA gene) and MG593271 (1431 bp *18S* rRNA gene).
- ***Vector*:** The vector potential of cf. *Haemaphysalis* sp. for *B. mackerrasorum* n. sp. is unknown.
- ***ZooBank registration*:** To comply with the regulations set out in article 8.5 of the amended 2012 version of the *International Code of Zoological Nomenclature* (ICZN) [24], details of the new species have been submitted to ZooBank. The Life Science Identifier (LSID) of the article is urn:lsid:zoobank.org:pub:D1B6E4E1-168C-488B-B809-381642900749. The LSID for the new name *Babesia mackerrasorum* n. sp. is urn:lsid:zoobank.org:act:BDEDBF3F-28B0-4A4B-B923-5A9EE47EF5B6.
- ***Etymology*:** This species is named after Dr Ian Murray Mackerras (1898–1980) and Dr Mabel Josephine Mackerras (1896–1971) in recognition of their contributions to Australian parasitology.

### Diagnosis

This organism is a species of *Babesia* (*s.s.*) that is genetically distinct from other described *Babesia* species and forms a clade with other *Babesia* (*s.s.*) species isolated from Australian marsupials and ticks (see above).

- **Order Eucoccidiorida Léger & Duboscq, 1910**
- **Suborder Adeleorina Léger, 1911**
- **Family Hepatozoidae Wenyon, 1926**
- **Genus** ***Hepatozoon*** **Miller, 1908**

### *Hepatozoon banethi* n. sp.

- ***Type-host*:** *Ixodes tasmani* Neumann (Acari: Ixodidae).
- ***Type-locality*:** Devonport, Tasmania, Australia.
- ***Other locality*:** Port Sorell, Tasmania, Australia.
- ***Type-material*:** Bisected tick, tissue extractions and genomic DNA were deposited at the Tasmanian Museum and Art Gallery, Hobart, Australia, under the accession numbers K4633 and K4637.
- ***Representative DNA sequences*:** DNA sequences were deposited in GenBank under the accessions MG758134 (303 bp *18S* rRNA gene), MG758135 (303 bp *18S* rRNA gene), MG758138 (303 bp *18S* rRNA gene), MG758133 (1656 bp *18S* rRNA gene), MG758135 (1668 bp *18S* rRNA gene), and MG758137 (1679 bp *18S* rRNA gene).
- ***Vector*:** The vector potential of *I. tasmani* for *H. banethi* n. sp. is unknown.
- ***ZooBank registration*:** To comply with the regulations set out in article 8.5 of the amended 2012 version of the *International Code of Zoological Nomenclature* (ICZN) [24], details of the new species have been submitted to ZooBank. The Life Science Identifier (LSID) of the article is urn:lsid:zoobank.org:pub:D1B6E4E1-168C-488B-B809-381642900749. The LSID for the new name *Hepatozoon banethi* n. sp. is urn:lsid:zoobank.org:act:B9AC9422-FB14-4BEA-B82C-7A7C901328EA.
- ***Etymology*:** This species is named for Professor Gad Baneth in recognition of his contributions to the field of vector-borne diseases, especially *Hepatozoon* infections in dogs.

### Diagnosis

This organism is a species of *Hepatozoon* that is genetically distinct from other described *Hepatozoon* species and forms a clade with other *Hepatozoon* species isolated from marsupials and ticks (see above).

### *Hepatozoon ewingi* n. sp.

- ***Type-host*:** *Haemaphysalis bancrofti* Nuttall and Warburton (Acari: Ixodidae).
- ***Type-locality*:** Eungai Creek, New South Wales, Australia.
- ***Type-material*:** Bisected tick, tissue extractions and genomic DNA were deposited at the Australian Museum, Sydney, Australia, under the accession numbers KS.128102.001-KS.128102.003.
- ***Representative DNA sequences*:** DNA sequences were deposited in GenBank under the accessions MG593274 (303 bp *18S* rRNA gene) and MG593275 (1680 bp *18S* rRNA gene).
- ***Vector*:** The vector potential of *H. bancrofti* for *H. ewingi* n. sp. is unknown.
- ***ZooBank registration*:** To comply with the regulations set out in article 8.5 of the amended 2012 version of the *International Code of Zoological Nomenclature* (ICZN) [24], details of the new species have been submitted to ZooBank. The Life Science Identifier (LSID) of the article is urn:lsid:zoobank.org:pub:D1B6E4E1-168C-488B-B809-381642900749. The LSID for the new name *Hepatozoon ewingi* n. sp. is urn:lsid:zoobank.org:act:4B0C5B4D-270F-4F3B-8160-1D8F8FA7CA3B.
- ***Etymology*:** This species is named after Professor Sidney Alton Ewing (1934–2018) who contributed more than fifty years of teaching and research to the field of veterinary parasitology.

### Diagnosis

This organism is a species of *Hepatozoon* that is genetically distinct from other described *Hepatozoon* species and forms a clade with other *Hepatozoon* species isolated from marsupials and ticks (see above).

- **Order Piroplasmida Wenyon, 1926**
- **Suborder Piroplasmorina Levine, 1971**
- **Family Theileriidae du Toit, 1918**
- **Genus** ***Theileria*** **Bettencourt, Franca & Borges, 1907**

### *Theileria apogeana* n. sp.

- ***Type-host*:** *Ixodes tasmani* Neumann (Acari: Ixodidae).
- ***Type-locality*:** Devonport, Tasmania, Australia.
- ***Type-material*:** Bisected tick, tissue extractions and genomic DNA were deposited at the Tasmanian Museum and Art Gallery, Hobart, Australia, under the accession number K4639.
- ***Representative DNA sequences*:** DNA sequences were deposited in GenBank under the accessions MG758126 (790 bp *18S* rRNA gene) and MG758116 (1480 bp *18S* rRNA gene).
- ***Vector*:** The vector potential of *I. tasmani* for *T. apogeana* n. sp. is unknown.
- ***ZooBank registration*:** To comply with the regulations set out in article 8.5 of the amended 2012 version of the *International Code of Zoological Nomenclature* (ICZN) [24], details of the new species have been submitted to ZooBank. The Life Science Identifier (LSID) of the article is urn:lsid:zoobank.org:pub:D1B6E4E1-168C-488B-B809-381642900749. The LSID for the new name *Theileria apogeana* n. sp. is urn:lsid:zoobank.org:act:808CAD4C-D259-40E3-B929-E308D23AADBD.
- ***Etymology*:** This species name is derived from the English adjective *apogean* which relates to the farthest or most distant point.

### Diagnosis

This organism is a species of *Theileria* Bettencourt that is genetically distinct from other described *Theileria* species and forms a clade with other *Theileria* species isolated from Australian marsupials and ticks (see above).

### *Theileria palmeri* n. sp.

- ***Type-host*:** *Ixodes tasmani* Neumann (Acari: Ixodidae).
- ***Type-locality*:** Port Sorell, Tasmania, Australia.
- ***Other locality:*** Devonport, Tasmania, Australia.
- ***Type-material*:** Tissue extractions and genomic DNA were deposited at the Tasmanian Museum and Art Gallery, Hobart, Australia, under the accession numbers K4632 and K4638.
- ***Representative DNA sequences*:** DNA sequences were deposited in GenBank under the accessions MG758125 (802 bp *18S* rRNA gene), MG758120 (1452 bp *18S* rRNA gene), and MG758113 (1506 bp *18S* rRNA gene).
- ***Vector*:** The vector potential of *I. tasmani* for *T. palmeri* n. sp. is unknown.
- ***ZooBank registration*:** To comply with the regulations set out in article 8.5 of the amended 2012 version of the *International Code of Zoological Nomenclature* (ICZN) [24], details of the new species have been submitted to ZooBank. The Life Science Identifier (LSID) of the article is urn:lsid:zoobank.org:pub:D1B6E4E1-168C-488B-B809-381642900749. The LSID for the new name *Theileria palmeri* n. sp. is urn:lsid:zoobank.org:act:6E82C4F6-D069-481F-9752-ADD852F42C57.
- ***Etymology*:** This species is named for Dr Dieter Palmer in recognition of his contributions to the field of parasitology.

### Diagnosis

This organism is a species of *Theileria* that is genetically distinct from other described *Theileria* species and forms a clade with other *Theileria* species isolated from Australian marsupials and ticks (see above).

### *Theileria paparinii* n. sp.

- ***Type-host*:** *Ixodes tasmani* Neumann (Acari: Ixodidae).
- ***Type-locality*:** Lower Wilmot, Tasmania, Australia.
- ***Other locality*:** Devonport, Tasmania, Australia.
- ***Type-material*:** Bisected tick, tissue extractions and genomic DNA were deposited at the Tasmanian Museum and Art Gallery, Hobart, Australia, under the accession numbers K4631 and K4635.
- ***Representative DNA sequences*:** DNA sequences were deposited in GenBank under the accessions MG758112 (309 bp *18S* rRNA gene), MG758117 (309 bp *18S* rRNA gene) and MG758115 (1496 bp *18S* rRNA gene).
- ***ZooBank registration*:** To comply with the regulations set out in article 8.5 of the amended 2012 version of the *International Code of Zoological Nomenclature* (ICZN) [24], details of the new species have been submitted to ZooBank. The Life Science Identifier (LSID) of the article is urn:lsid:zoobank.org:pub:D1B6E4E1-168C-488B-B809-381642900749. The LSID for the new name *Theileria paparinii* n. sp. is urn:lsid:zoobank.org:act:0BD6DD5B-5453-416E-8E81-3EEA38DB5FCE.
- ***Etymology*:** This species is named for Dr Andrea Paparini, Murdoch University, Australia, in recognition of his contributions to Australian piroplasm research.

### Diagnosis

This organism is a species of *Theileria* that is genetically distinct from other described *Theileria* species and forms a clade with other *Theileria* species isolated from Australian marsupials and ticks (see above).

### *Theileria worthingtonorum* n. sp.

- ***Type-host*:** *Ixodes tasmani* Neumann (Acari: Ixodidae).
- ***Type-locality*:** Port Sorell, Tasmania, Australia.
- ***Type-material*:** Tissue extractions and genomic DNA were deposited at the Tasmanian Museum and Art Gallery, Hobart, Australia, under the accession numbers K4634 and K4636.
- ***Representative DNA sequences*:** DNA sequences were deposited in GenBank under the accessions MG758118 (310 bp *18S* rRNA gene), MG758119 (310 bp *18S* rRNA gene), MG758121 (1497 bp *18S* rRNA gene) and MG758114 (1504 bp *18S* rRNA gene).
- ***Vector*:** The vector potential of *I. tasmani* for *T. worthingtonorum* n. sp. is unknown.
- ***ZooBank registration*:** To comply with the regulations set out in article 8.5 of the amended 2012 version of the *International Code of Zoological Nomenclature* (ICZN) [24], details of the new species have been submitted to ZooBank. The Life Science Identifier (LSID) of the article is urn:lsid:zoobank.org:pub:D1B6E4E1-168C-488B-B809-381642900749. The LSID for the new name *Theileria worthingtonorum* n. sp. is urn:lsid:zoobank.org:act:89A747A5-5D91-47A1-84EB-AF01236145B0.
- ***Etymology*:** This species is named for the first author’s grandparents Mr Peter Ross Worthington and Mrs Dawn Rose Worthington.

### Diagnosis

This organism is a species of *Theileria* that is genetically distinct from other described *Theileria* species and forms a clade with other *Theileria* species isolated from Australian marsupials and ticks (see above).

## Discussion

This study is the first to investigate, on a national scale, apicomplexan parasites carried by ticks that parasitise companion animals in Australia. Although we hypothesised that species of endemic piroplasms would be detected in ticks parasitising dogs, cats and horses, the discovery of nine novel apicomplexan species was unexpected. The low pairwise identities and distinct phylogenetic groupings of the novel *18S* sequences to the most closely related described species supports the new species classifications. This investigation used an approach similar to that used by Schnittger et al. [25] to taxonomically assign the sequences to species. All *Babesia* and *Theileria* species sequenced in this study were ≤ 96.4% and ≤ 93.3% similar to the most closely related named species, respectively, with the exception of *B. mackerrasorum*, which was 98.3% similar to *B. macropus* (JQ437265)*.* The highest pairwise similarity for the novel *Hepatozoon* species compared to named *Hepatozoon* species was 96.6%, which is less than the pairwise identity of the recently described *Hepatozoon musa* Borges-Nojosa, 2017 to its most closely related described species (~99% similar) [26].

Moreover, the new species assignments are further supported by the distinct phylogenetic groupings of the novel species to named species (Figs. 1 and 3). *Babesia mackerrasorum* n. sp. and *B. lohae* n. sp. are most closely related to *Babesia* spp. that have been isolated previously from Australian marsupials and ticks from marsupials (Figs. 1 and 2). *Babesia mackerrasorum* n. sp. clustered separately with a longer branch length to *B. macropus* (JQ437265 and JQ437266) isolated from eastern grey kangaroos (*M. giganteus*) in NSW and QLD. Additionally, the intraspecific genetic variation of *B. macropus* at the *18S* gene is 0.2% [27, 28], and as the interspecific genetic variation at the *18S* gene between *B. macropus* and *B. mackerrasorum* n. sp. was 1.7%, this also suggests that they are different species. *Babesia lohae* n. sp. is 100% similar to an unnamed *Babesia* sp. sequence (MG251436) derived from a common marsupial tick (*I. tasmani*), collected from a common brushtail possum (*T. vulpecula*), also in QLD (unpublished), and thus it is possible that the brushtail possum is a native reservoir host of *B. lohae* n. sp., and although a number of *Babesia* species have been identified in the blood of native marsupials [27–30], no studies have yet investigated brushtail possums for *Babesia* spp.

The novel *Theileria* species also phylogenetically clustered with *18S* sequences derived from native marsupials (Figs. 1 and 2), suggesting that the *Theileria* species identified in *I. tasmani* have marsupial hosts. *Theileria paparinii* n. sp. is most closely related to, but distinct from, the previously described *Theileria penicillata* Clark and Spencer 2007 and *Theileria brachyuri* Clark and Spencer 2007 that were isolated from woylies and quokkas, respectively, in WA [31, 32]. *Theileria worthingtonorum* n. sp. is most closely related to *Theileria fuliginosa* Clark and Spencer 2007 from the western grey kangaroo (*Macropus fuliginosus* Desmarest) in WA [31]. *Ixodes tasmani* has been described as catholic in its feeding habits having been recorded on 42 host species, including marsupials, monotremes, rodents and domestic animals [8]. It is likely that there are multiple native host species for the novel *Theileria* species, and for *H. banethi* n. sp. that was also identified in *I. tasmani*. Both *H. ewingi* n. sp. (isolated from *H. bancrofti*, wallaby tick, which feeds on bandicoots, possums, macropods and other marsupials [9]) and *H. banethi* n. sp. group with *Hepatozoon* spp. sequenced from *D. gliroides* [33], a marsupial from Chile. This again lends weight to the suggestion that these new Australian *Hepatozoon* species have native marsupial hosts. Other *Hepatozoon* species have been described from Australian wildlife, including reptiles [34–37], ticks [38] and bandicoots (*Perameles* Geoffroy Saint-Hilaire spp. and *Isoodon* Desmarest spp.) [37], the latter of which was < 97% similar to the *Hepatozoon* species from this study (data not shown).

Although many apicomplexan species are difficult to morphologically differentiate between or are indistinguishable at the species level [38], there are distinct morphological differences between families and genera. Therefore, morphological characterisations, with additional genetic characterisations, are required to confirm the family and genus of cf. Sarcocystidae gen. sp., although based on the pairwise distances and phylogenetic reconstructions, this sequence certainly represents a novel species.

This study has demonstrated that the use of conventional PCR and Sanger sequencing for characterising apicomplexans in ticks is limited due to the identification of co-infections of piroplasms and *Hepatozoon* species, and co-infections of multiple *Theileria* species (outlined in Table 4). To more comprehensively identify co-infections in ticks, a next-generation sequencing (NGS) approach could be used, which has been shown to be a useful technique for identifying *Trypanosoma* Gruby, 1843 spp. in ticks [39]. There are likely other protozoans in native ticks that remain to be discovered, and future studies could aim to extend this NGS approach to protozoans in general, which would greatly improve the speed and lower the cost for studies that aim to broadly screen for protists.

The prevalence of novel apicomplexan species detected in this study was relatively high (1.3%; 9/711; 95% CI: 0.6–2.4%), which reflects that apicomplexan species in ticks and their hosts in Australia has been greatly understudied to date. Previous studies that have investigated the prevalence of piroplasms and *Hepatozoon* spp. in Australian wildlife have reported these Apicomplexa to be highly prevalent in their hosts. For example, *T. penicillata* has been reported in *Bettongia penicillata* Gray in WA at a prevalence of 80.4% (123/153) [32]. *Hepatozoon* sp. has been found in 34.1% (15/44) of *I. tasmani* ticks collected from Tasmanian devils (*Sarcophilus harrisii* Boitard) [40], while the prevalence of *H. banethi* n. sp. in *I. tasmani* ticks tested in this study was considerably lower (5.1%; 3/59; 95% CI: 1.1–14.1%). *Hepatozoon* sp. has been detected in the southern brown bandicoot (*Isoodon obesulus* Shaw) in WA in 58.1% (18/31) of samples [37], and a high prevalence of *Hepatozoon* spp. has also been reported in reptiles. For example, a study by Jakes et al. [34] detected *Hepatozoon boigae* Mackerras, 1961 in 29% of 97 brown tree snakes (*Boiga irregularis* Merrem), and *Hepatozoon* spp. has been detected in 35.6% of 35 blood samples from water pythons (*Liasis fuscus* Peters) with 57.7% of 187 ticks collected from *L. fuscus* also positive for *Hepatozoon* spp. [38], and another study has detected *Hepatozoon* spp. in 100% of 100 blood samples from *L. fuscus* [16].

It is not surprising that *B. vogeli* and *T. orientalis* genotype 2 (Ikeda) were identified in *R. sanguineus* and *H. longicornis*, respectively. *Rhipicephalus sanguineus* is a competent vector of *B. vogeli* [5, 6] and *H. longicornis* is likely a vector of *T. orientalis* genotype Ikeda [41]. The finding of *H. canis*, a tick-borne pathogen of dogs that, to our knowledge, has not been recorded in Australia previously, was unanticipated. Of note is that *H. canis* was detected in a paralysis tick, *I. holocyclus*, which has not been associated with *H. canis* before. Studies have shown that *R. sanguineus* is a vector [42] and *H. longicornis* is a putative vector [43] of *H. canis*. These species are present in Australia, and although no *R. sanguineus* (0/183; 95% CI: 0–2%) or *H. longicornis* (0/75; 95% CI: 0–4.8%) ticks examined in the present study contained *H. canis* DNA, the prevalence of *H. canis* in ticks collected from dogs in endemic areas has been reported to be as low as 1.5% (4/267) [44]. It is also possible that the *I. holocyclus* tick (which was engorged with the host’s blood) ingested a *H. canis*-infected blood meal from the host. This is not the first instance of an exotic tick-borne pathogen of companion animals in Australia; in the 1970s, there was a brief incursion of *Theileria equi* in imported horses, which caused localised outbreaks of equine piroplasmosis [45–47]. Without this broad investigation for piroplasms and *Hepatozoon* spp. in ticks, it is likely that *H. canis* would have remained undetected, which emphasises the need for ongoing surveillance of tick-borne pathogens across the country. Australian biosecurity authorities have been advised of this finding and an investigation into the potential sources and dissemination of this presumed incursion of *H. canis* is under way.

## Conclusion

This investigation of more than 700 ticks in Australia has led to the discovery of nine new apicomplexans, the exotic canine pathogen *H. canis*, and detected the endemic tick-borne pathogens *B. vogeli* and *T. orientalis* genotype Ikeda. Future studies are required to establish the host range and the vector competence of ticks for the newly described apicomplexans as these haemoprotozoans could represent an infectious disease threat to companion animal species.

## Additional files

Additional file 1: **Table S1.** Collection locations of ticks from dogs, cats and horses that were screened for piroplasms and *Hepatozoon* spp. (PDF 261 kb)

Additional file 2: **Table S2.** Summary of PCR screening and NCBI BLAST results for apicomplexan sequences obtained in this study. (PDF 200 kb)

Additional file 3: **Table S3.** Prevalence of Apicomplexa species Australia-wide and in each state and territory summarised for all tick species from all hosts, individual tick species from all hosts, and individual tick species from dogs, cats and horses. (XLSX 35 kb)

Additional file 4: **Table S4.** Pairwise genetic distance matrix of *18S* sequences similarities (%) from novel piroplasms and *Hepatozoon* spp. described in this study. (PDF 202 kb)

## Abbreviations

*18S*: *18S* ribosomal RNA gene
BIC: Bayesian Information Criterion
ExCs: Extraction reagent blank controls
MPSP: Major piroplasm surface protein
nr/nt: Non-redundant nucleotide
NSW: New South Wales
NT: Northern Territory
NTCs: No-template controls
pp: Posterior probabilities
QLD: Queensland
SA: South Australia
SD: Standard deviation
SNPs: Single nucleotide polymorphisms
Tann: Annealing temperature
TAS: Tasmania
VIC: Victoria
WA: Western Australia
