# Supplementary material for: Endemic, exotic and novel apicomplexan parasites detected during a national study of ticks from companion animals in Australia
Source: Parasit Vectors. 2018 Mar 20;11:197. doi: 10.1186/s13071-018-2775-y (PMC5859549; doi:10.1186/s13071-018-2775-y)
Supplement: Supplementary file 1 — Table S1. Collection locations of ticks from dogs, cats and horses that were screened for piroplasms and Hepatozoon spp. (PDF 261 kb) [file 13071_2018_2775_MOESM1_ESM.pdf]

Additional file 1: Table S1. Collection locations of ticks from dogs, cats and horses that were screened for piroplasms and *Hepatozoon* spp.

Tick species	Instar	Location	New South Wales	Northern Territory	Queensland	South Australia	Tasmania	Victoria	Western Australia	Grand Total
Amblyomma triguttatum triguttatum	Female	Bullsbrook	0	0	0	0	0	0	1	1
		Gidgegannup	0	0	0	0	0	0	9	9
		Perth	0	0	0	0	0	0	2	2
		Rowan Wood	1	0	0	0	0	0	0	1
		Tanja	2	0	0	0	0	0	0	2
		Woodridge	0	0	0	0	0	0	1	1
		Wyneden	1	0	0	0	0	0	0	1
		Yelarbon	0	0	2	0	0	0	0	2
	Nymph	Gidgegannup	0	0	0	0	0	0	1	1
		Mount Helena	0	0	0	0	0	0	3	3
		Tanja	1	0	0	0	0	0	0	1
	**Subtotal**		**5**	**0**	**2**	**0**	**0**	**0**	**17**	**24**
Bothriocroton sp.	Female	Caldermeade	0	0	0	0	0	1	0	1
	Male	Willow Grove	0	0	0	0	0	1	0	1
	Larva	Dandenong	0	0	0	0	0	4	0	4
	**Subtotal**		**0**	**0**	**0**	**0**	**0**	**6**	**0**	**6**
Haemaphysalis bancrofti	Female	Eungai Creek	2	0	0	0	0	0	0	2
		Missabotti	1	0	0	0	0	0	0	1
	Male	Currumbin Valley	0	0	1	0	0	0	0	1
		Eungai Creek	1	0	0	0	0	0	0	1
	**Subtotal**		**4**	**0**	**1**	**0**	**0**	**0**	**0**	**5**
Haemaphysalis lagostrophi	Female	Atherton	0	0	1	0	0	0	0	1
	**Subtotal**		**0**	**0**	**1**	**0**	**0**	**0**	**0**	**1**
Haemaphysalis longicornis	Female	Bellingen	7	0	0	0	0	0	0	7
		Bowraville	5	0	0	0	0	0	0	5
		Byabarra	1	0	0	0	0	0	0	1
		Currumbin Valley	0	0	2	0	0	0	0	2
		Eungai Creek	2	0	0	0	0	0	0	2
		Missabotti	17	0	0	0	0	0	0	17
		Sancrox	1	0	0	0	0	0	0	1
		Sydney	1	0	0	0	0	0	0	1
		Tanja	1	0	0	0	0	0	0	1
		Verona	5	0	0	0	0	0	0	5
		Wyrallah	1	0	0	0	0	0	0	1
	Male	Bellingen	1	0	0	0	0	0	0	1
	Nymph	Bellingen	2	0	0	0	0	0	0	2
		Brisbane	0	0	3	0	0	0	0	3
		Currumbin Valley	0	0	1	0	0	0	0	1
		Eungai Creek	1	0	0	0	0	0	0	1
		Missabotti	21	0	0	0	0	0	0	21
		Thumb Creek	1	0	0	0	0	0	0	1
		Verona	2	0	0	0	0	0	0	2
	**Subtotal**		**69**	**0**	**6**	**0**	**0**	**0**	**0**	**75**
Haemaphysalis sp.	Female	Tanja	1	0	0	0	0	0	0	1
	Male	Tanja	2	0	0	0	0	0	0	2
	**Subtotal**		**3**	**0**	**0**	**0**	**0**	**0**	**0**	**3**
Ixodes cornuatus	Female	Devonport	0	0	0	0	3	0	0	3
	Nymph	Devonport	0	0	0	0	1	0	0	1
		Launceston	0	0	0	0	1	0	0	1
		Lower Wilmot	0	0	0	0	3	0	0	3
		Mallacoota	0	0	0	0	0	2	0	2
	**Subtotal**		**0**	**0**	**0**	**0**	**8**	**2**	**0**	**10**
Ixodes hirsti	Female	Devonport	0	0	0	0	1	0	0	1
	**Subtotal**		**0**	**0**	**0**	**0**	**1**	**0**	**0**	**1**
Ixodes holocyclus	Female	Atherton	0	0	17	0	0	0	0	17
		Beechmont	0	0	1	0	0	0	0	1
		Bellingen	9	0	0	0	0	0	0	9
		Bowraville	5	0	0	0	0	0	0	5
		Brinsmead	0	0	12	0	0	0	0	12
		Brisbane	0	0	2	0	0	0	0	2
		Byangum	1	0	0	0	0	0	0	1
		Byron Bay	6	0	0	0	0	0	0	6
		Calder	0	0	0	0	1	0	0	1
		Callala Bay	2	0	0	0	0	0	0	2
		Charlestown	5	0	0	0	0	0	0	5
		Coffs Harbour	3	0	0	0	0	0	0	3
		Culburra Beach	3	0	0	0	0	0	0	3
		Cooroy	0	0	1	0	0	0	0	1
		Currumbin Valley	0	0	7	0	0	0	0	7
		Devonport	0	0	0	0	1	0	0	1
		Eungai Creek	6	0	0	0	0	0	0	6
		Greenwich	1	0	0	0	0	0	0	1
		Hawkesbury River	1	0	0	0	0	0	0	1

		Image Flat	0	0	3	0	0	0	0	3
		Kempsey	1	0	0	0	0	0	0	1
		Killarney Heights	2	0	0	0	0	0	0	2
		Kuranda	0	0	6	0	0	0	0	6
		Lane Cove	1	0	0	0	0	0	0	1
		Lindfield	5	0	0	0	0	0	0	5
		Mackay	0	0	10	0	0	0	0	10
		Marsfield	1	0	0	0	0	0	0	1
		Medowie	1	0	0	0	0	0	0	1
		Merimbula	5	0	0	0	0	0	0	5
		Missabotti	5	0	0	0	0	0	0	5
		Mona Vale	10	0	0	0	0	0	0	10
		Moruya	12	0	0	0	0	0	0	12
		Murrah	1	0	0	0	0	0	0	1
		Nambour	0	0	2	0	0	0	0	2
		Narooma	5	0	0	0	0	0	0	5
		North Bega	6	0	0	0	0	0	0	6
		Northbridge	1	0	0	0	0	0	0	1
		Pambula	3	0	0	0	0	0	0	3
		Park Ridge	0	0	6	0	0	0	0	6
		Peats Ridge	2	0	0	0	0	0	0	2
		Roseville	1	0	0	0	0	0	0	1
		Sarina	0	0	8	0	0	0	0	8
		Seaforth	10	0	0	0	0	0	0	10
		St Ives	1	0	0	0	0	0	0	1
		Stanmore	1	0	0	0	0	0	0	1
		Tambellup	0	0	0	0	0	0	1	1
		Tanja	1	0	0	0	0	0	0	1
		Thumb Creek	1	0	0	0	0	0	0	1
		Trinity Beach	0	0	7	0	0	0	0	7
		Tully	0	0	22	0	0	0	0	22
		Turramurra	13	0	0	0	0	0	0	13
		Wagga Wagga	1	0	0	0	0	0	0	1
		Wollstonecraft	1	0	0	0	0	0	0	1
		Wyoming	4	0	0	0	0	0	0	4
		Wyrallah	4	0	0	0	0	0	0	4
		Yeppoon	0	0	4	0	0	0	0	4
	Male	Atherton	0	0	1	0	0	0	0	1
		Beechmont	0	0	1	0	0	0	0	1
		Brisbane	0	0	1	0	0	0	0	1
		Byron Bay	1	0	0	0	0	0	0	1
		Currumbin Valley	0	0	1	0	0	0	0	1
		Eungai Creek	2	0	0	0	0	0	0	2
		Killarney Heights	1	0	0	0	0	0	0	1
		Missabotti	33	0	0	0	0	0	0	33
		Moruya	1	0	0	0	0	0	0	1
		North Bega	1	0	0	0	0	0	0	1
		Turramurra	1	0	0	0	0	0	0	1
		Wollongbar	1	0	0	0	0	0	0	1
	Nymph	Atherton	0	0	1	0	0	0	0	1
		Brinsmead	0	0	1	0	0	0	0	1
		Brisbane	0	0	3	0	0	0	0	3
		Devonport	0	0	0	0	1	0	0	1
		Eungai Creek	1	0	0	0	0	0	0	1
		Gulmarrad	3	0	0	0	0	0	0	3
		Killarney Heights	1	0	0	0	0	0	0	1
		Kuranda	0	0	3	0	0	0	0	3
		Mackay	0	0	1	0	0	0	0	1
		Missabotti	7	0	0	0	0	0	0	7
		North Bega	2	0	0	0	0	0	0	2
		Sarina	0	0	1	0	0	0	0	1
		Tanja	1	0	0	0	0	0	0	1
		Trinity Beach	0	0	1	0	0	0	0	1
		Turramurra	4	0	0	0	0	0	0	4
		Wollongbar	7	0	0	0	0	0	0	7
	**Subtotal**		**208**	**0**	**123**	**0**	**3**	**0**	**1**	**335**
Ixodes myrmecobii	Female	Esperance	0	0	0	0	0	0	4	4
		Hopetoun Beach	0	0	0	0	0	0	1	1
	**Subtotal**		**0**	**0**	**0**	**0**	**0**	**0**	**5**	**5**
Ixodes tasmani	Female	Dandenong	0	0	0	0	0	2	0	2
		Devonport	0	0	0	0	25	0	0	25
		East Devonport	0	0	0	0	1	0	0	1
		Kuranda	0	0	1	0	0	0	0	1
		Lower Wilmot	0	0	0	0	7	0	0	7
		Merseylea	0	0	0	0	1	0	0	1
		Northdown	0	0	0	0	1	0	0	1
		Port Sorell	0	0	0	0	4	0	0	4
		Sarina	0	0	2	0	0	0	0	2
		Sassafras	0	0	0	0	5	0	0	5

		Seaforth	1	0	0	0	0	0	0	1
	Male	Northdown	0	0	0	0	1	0	0	1
	Nymph	Devonport	0	0	0	0	2	0	0	2
		Lower Wilmot	0	0	0	0	1	0	0	1
		Northdown	0	0	0	0	1	0	0	1
		Sassafras	0	0	0	0	1	0	0	1
		Trafalgar	0	0	0	0	0	1	0	1
	Larva	Devonport	0	0	0	0	3	0	0	3
	**Subtotal**		**1**	**0**	**2**	**0**	**53**	**3**	**0**	**59**
Rhipicephalus australis	Nymph	Kuranda	0	0	2	0	0	0	0	2
		Sarina	0	0	1	0	0	0	0	1
	**Subtotal**		**0**	**0**	**3**	**0**	**0**	**0**	**0**	**3**
Rhipicephalus sanguineus	Female	Atherton	0	0	1	0	0	0	0	1
		Bagot	0	3	0	0	0	0	0	3
		Broome	0	0	0	0	0	0	1	1
		Cable Beach	0	0	0	0	0	0	3	3
		Carnarvon	0	0	0	0	0	0	4	4
		Coober Pedy	0	0	0	4	0	0	0	4
		Halls Creek	0	0	0	0	0	0	3	3
		Kalumburu	0	0	0	0	0	0	2	2
		Karratha	0	0	0	0	0	0	1	1
		Katherine	0	6	0	0	0	0	0	6
		Kuranda	0	0	1	0	0	0	0	1
		Kurnangki Community	0	0	0	0	0	0	2	2
		Lake Nash (Alpurrurulam Community)	0	1	0	0	0	0	0	1
		Mackay	0	0	1	0	0	0	0	1
		Medowie	3	0	0	0	0	0	0	3
		Mindarie	0	0	0	0	0	0	1	1
		Mutitjulu Community	0	4	0	0	0	0	0	4
		Oodnadatta	0	0	0	10	0	0	0	10
		Palmerston Indigenous Village	0	3	0	0	0	0	0	3
		Palmerston	0	1	0	0	0	0	0	1
		Parap	0	1	0	0	0	0	0	1
		Pinjar	0	0	0	0	0	0	1	1
		Sarina	0	0	3	0	0	0	0	3
		Torres Strait	0	0	17	0	0	0	0	17
		Trinity Beach	0	0	1	0	0	0	0	1
		Yeppoon	0	0	1	0	0	0	0	1
		Yuelamu (Mt Allan)	0	1	0	0	0	0	0	1
	Male	Atherton	0	0	1	0	0	0	0	1
		Bagot	0	2	0	0	0	0	0	2
		Broome	0	0	0	0	0	0	1	1
		Cable Beach	0	0	0	0	0	0	3	3
		Carnarvon	0	0	0	0	0	0	3	3
		Coober Pedy	0	0	0	6	0	0	0	6
		Halls Creek	0	0	0	0	0	0	2	2
		Kalumburu	0	0	0	0	0	0	3	3
		Katherine	0	3	0	0	0	0	0	3
		Kuranda	0	0	1	0	0	0	0	1
		Kurnangki Community	0	0	0	0	0	0	2	2
		Lake Nash (Alpurrurulam Community)	0	1	0	0	0	0	0	1
		Marble Bar	0	0	0	0	0	0	1	1
		Mutitjulu Community	0	4	0	0	0	0	0	4
		Nyirripi Community	0	1	0	0	0	0	0	1
		Oodnadatta	0	0	0	10	0	0	0	10
		Palmerston Indigenous Village	0	1	0	0	0	0	0	1
		Parap	0	2	0	0	0	0	0	2
		Sarina	0	0	1	0	0	0	0	1
		Trinity Beach	0	0	1	0	0	0	0	1
		Yeppoon	0	0	1	0	0	0	0	1
		Yuendumu	0	1	0	0	0	0	0	1
	Nymph	Bagot	0	1	0	0	0	0	0	1
		Cable Beach	0	0	0	0	0	0	2	2
		Coober Pedy	0	0	0	1	0	0	0	1
		Halls Creek	0	0	0	0	0	0	1	1

		Kulaluk	0	1	0	0	0	0	0	1
		Kalumburu	0	0	0	0	0	0	9	9
		Karratha	0	0	0	0	0	0	2	2
		Katherine	0	5	0	0	0	0	0	5
		Lake Nash (Alpurrurulam Community)	0	1	0	0	0	0	0	1
		Murdoch	0	0	0	0	0	0	1	1
		Mutitjulu Community	0	2	0	0	0	0	0	2
		Oodnadatta	0	0	0	14	0	0	0	14
		Palmerston	0	1	0	0	0	0	0	1
		Sarina	0	0	2	0	0	0	0	2
		Yeppoon	0	0	1	0	0	0	0	1
		Yuendumu	0	1	0	0	0	0	0	1
	Larva	Kalumburu	0	0	0	0	0	0	1	1
		Katherine	0	1	0	0	0	0	0	1
		Mindarie	0	0	0	0	0	0	1	1
		Oodnadatta	0	0	0	3	0	0	0	3
		Palmerston Indigenous Village	0	1	0	0	0	0	0	1
		Yuendumu	0	1	0	0	0	0	0	1
	**Subtotal**		**3**	**50**	**33**	**48**	**0**	**0**	**50**	**184**
**Grand total**			**293**	**50**	**171**	**48**	**65**	**11**	**73**	**711**
