# Supplementary material for: Endemic, exotic and novel apicomplexan parasites detected during a national study of ticks from companion animals in Australia
Source: Parasit Vectors. 2018 Mar 20;11:197. doi: 10.1186/s13071-018-2775-y (PMC5859549; doi:10.1186/s13071-018-2775-y)
Supplement: Supplementary file 2 — Table S2. Summary of PCR screening and NCBI BLAST results for apicomplexan sequences obtained in this study. (PDF 200 kb) [file 13071_2018_2775_MOESM2_ESM.pdf]

Additional file 2: Table S2. Summary of PCR screening results for piroplasms and *Hepatozoon* spp. with various primer sets and the sequencing and NCBI BLAST results

Tick species	Instar	Host	Tick gDNA sample name	Host's name	Collection location	Primers	PCR results	Apicomplexa species	GenBank accession number	ZooBank nomenclatural act number	Length (bp)	Top NCBI blast hit	Top NCBI blast hit accession numbers	Pairwise distances (% sequence similarity)
Haemaphysalis bancrofti (*n* = 1)	Male	Horse	HBM1	H-HBM1	Eungai Creek, NSW	18SApiF/18SApiR	Positive	*Hepatozoon ewingi* n. sp.	MG593274	4B0C5B4D-270F-4F3B-8160-1D8F8FA7CA3B	303	*Hepatozoon* sp. ex rattlesnake BR-2012 strain Hep_3 18S ribosomal RNA gene, partial sequence	KC342523	97.0
						HAM-1F/HPF-2R			MG593275		1680	*Hepatozoon* sp. DG1 small subunit ribosomal RNA gene, partial sequence	FJ719813	96.3
						BTF2/BTR2	Negative	N/A*	N/A	N/A	N/A	N/A	N/A	N/A
Haemaphysalis longicornis (*n* = 3)	Nymphs	Dogs	HLN1	D-HLN1	Missabotti, NSW	18SApiF/18SApiR	Positive	*Theileria orientalis* genotype Ikeda	MG571582		309	*Theileria* sp. clone N11 18S ribosomal RNA gene, partial sequence	KY197711	100
						Ts-U/TS-R			MG758109		835	*Theileria* sp. Luzon_2 gene for major piroplasm surface protein, partial cds, clone: *Theileria*_spp._Luzon_2	LC007096	100
						HepF300/Hep900	Negative	N/A	N/A		N/A	N/A	N/A	N/A
						HEMO1/HEMO2								
			HLN2	D-HLN2	Verona, NSW	18SApiF/18SApiR	Positive	*Theileria orientalis* genotype Ikeda	MG571581		309	*Theileria* sp. clone N11 18S ribosomal RNA gene, partial sequence	KY197711	100
						Ts-U/TS-R			MG758110		835	*Theileria* sp. Luzon_2 gene for major piroplasm surface protein, partial cds, clone: *Theileria*_spp._Luzon_2	LC007096	100
						HepF300/Hep900	Negative	N/A	N/A		N/A	N/A	N/A	N/A
						HEMO1/HEMO2								
			HLN3	D-HLN3	Missabotti, NSW	18SApiF/18SApiR	Mixed[a]							
						BTF2/BTR2	Positive	*Theileria orientalis* genotype Ikeda	MG571580		778	*Theileria* sp. clone N11 18S ribosomal RNA gene, partial sequence	KY197711	100
						Ts-U/TS-R			MG758111		835	*Theileria* sp. Luzon_2 gene for major piroplasm surface protein, partial cds, clone: *Theileria*_spp._Luzon_2	LC007096	100
						HepF300/Hep900	Negative	N/A	N/A		N/A	N/A	N/A	N/A
						HEMO1/HEMO2								
cf. *Haemaphysalis* sp. (*n* = 1)	Male	Horse	HspM1	H-HspM1	Tanja, NSW	18SApiF/18SApiR	Positive	*Babesia mackerrasorum* n. sp.	MG593276	BDEDBF3F-28B0-4A4B-B923-5A9EE47EF5B6	299	*Babesia occultans* isolate Trender1 18S ribosomal RNA gene, partial sequence	KP745626	98.0
						Nbab_1F/18SApiR	Positive		MG593271		1431	*Babesia* sp. ALT-2012 strain 3 NSW 18S ribosomal RNA gene, partial sequence	JQ437265	98.3
						HepF300/Hep900	Negative	N/A	N/A	N/A	N/A	N/A	N/A	N/A
						HEMO1/HEMO2								
Ixodes holocyclus (*n* = 2)	Females	Cat	IHF1	C-IHF1	Park Ridge, QLD	18SApiF/18SApiR	Positive	*Babesia lohae* n. sp.	MG593273	7526D345-A3A5-4483-8AB6-599F76F0DA32	299	*Babesia* sp. 33 18S ribosomal RNA gene, partial sequence	JQ682876	98.3
						Nbab_1F/18SApiR			MG593272		1430	*Babesia* sp. isolate BP7 small subunit ribosomal RNA gene, partial sequence	MG251436	100
						HepF300/Hep900	Negative	N/A	N/A	N/A	N/A	N/A	N/A	N/A
						HEMO1/HEMO2								
		Dog	IHF2	D-IHF2	Sarina, QLD	18SApiF/18SApiR	Positive	*Hepatozoon canis*	MG062865		303	*Hepatozoon canis* isolate 3470 18S ribosomal RNA gene, partial sequence	KX712128	100
						HepF300/Hep900			MG758124		1409	*Hepatozoon canis* isolate 1 18S ribosomal RNA gene, partial sequence	KX712124	99.9
						HEMO1/HEMO2								
						BTF2/BTR2	Negative	N/A	N/A		N/A	N/A	N/A	N/A
Ixodes tasmani (*n* = 8)	Females	Dogs	ITF1	D-ITF1	Devonport, TAS	18SApiF/18SApiR	Positive	*Theileria paparinii* n. sp.	MG758112	0BD6DD5B-5453-416E-8E81-3EEA38DB5FCE	309	*Theileria* sp. K1 18S ribosomal RNA gene, partial sequence	JQ682879	97.1
						Nbab_1F/18SApiR	Positive	*Theileria palmeri* n. sp.	MG758113	6E82C4F6-D069-481F-9752-ADD852F42C57	1506	*Theileria* sp. 18S ribosomal RNA gene, partial sequence	MF576261	95.5
						HepF300/Hep900	Negative	N/A	N/A	N/A	N/A	N/A	N/A	N/A
						HEMO1/HEMO2								
			ITF2	D-ITF2	Devonport, TAS	18SApiF/18SApiR	Positive	*Hepatozoon banethi* n. sp.	MG758134	B9AC9422-FB14-4BEA-B82C-7A7C901328EA	303	*Hepatozoon* sp. DJH-2014c isolate CHUFCL4282 18S ribosomal RNA gene, partial sequence	KM234615	99.7
						HAM-1F/HPF-2R			MG758133		1656	*Hepatozoon* sp. DG1 small subunit ribosomal RNA gene, partial sequence	FJ719813	98.0
						BTF2/BTR2	Negative	N/A	N/A	N/A	N/A	N/A	N/A	N/A
			ITF3	D-ITF3	Port Sorell, TAS	18SApiF/18SApiR	Positive	*Theileria worthingtonorum* n. sp.	MG758118	89A747A5-5D91-47A1-84EB-AF01236145B0	310	*Theileria* sp. 18S ribosomal RNA gene, partial sequence	MF576261	99.0
						Nbab_1F/18SApiR			MG758114		1504	*Theileria* sp. 18S ribosomal RNA gene, partial sequence	MF576261	98.3
						HepF300/Hep900	Negative	N/A	N/A	N/A	N/A	N/A	N/A	N/A
						HEMO1/HEMO2								
			ITF4	D-ITF4	Lower Wilmot, TAS	18SApiF/18SApiR	Positive	*Theileria paparinii* n. sp.	MG758117	0BD6DD5B-5453-416E-8E81-3EEA38DB5FCE	309	*Theileria* sp. K1 18S ribosomal RNA gene, partial sequence	JQ682879	97.1
						Nbab_1F/18SApiR			MG758115		1496	*Theileria* sp. K1 18S ribosomal RNA gene, partial sequence	JQ682879	98.9
						HepF300/Hep900	Negative	N/A	N/A	N/A	N/A	N/A	N/A	N/A
						HEMO1/HEMO2								
			ITF5	D-ITF5	Lower Wilmot, TAS	18SApiF/18SApiR	Positive	*Theileria worthingtonorum* n. sp.	MG758119	89A747A5-5D91-47A1-84EB-AF01236145B0	310	*Theileria* sp. 18S ribosomal RNA gene, partial sequence	MF576261	99.0
						Nbab_1F/18SApiR			MG758121		1497	*Theileria* sp. 18S ribosomal RNA gene, partial sequence	MF576261	98.3
						HepF300/Hep900	Negative	N/A	N/A	N/A	N/A	N/A	N/A	N/A
						HEMO1/HEMO2								
			ITF6	D-ITF6	Port Sorell, TAS	18SApiF/18SApiR	Positive	*Hepatozoon banethi* n. sp.	MG758135	B9AC9422-FB14-4BEA-B82C-7A7C901328EA	303	*Hepatozoon musa* isolate Co1031 18S ribosomal RNA gene, partial sequence	KX880079	99.7
						HAM-1F/HPF-2R			MG758136		1668	*Hepatozoon* sp. DG1 small subunit ribosomal RNA gene, partial sequence	FJ719813	98.0
						BTF2/BTR2		*Theileria palmeri* n. sp.	MG758125	6E82C4F6-D069-481F-9752-ADD852F42C57	802	*Theileria* sp. 18S ribosomal RNA gene, partial sequence	MF576261	95.5
						Nbab_1F/18SApiR			MG758120		1452	*Theileria* sp. 18S ribosomal RNA gene, partial sequence	MF576261	95.5
			ITF7	D-ITF7	Devonport, TAS	18SApiF/18SApiR		*Hepatozoon banethi* n. sp.	MG758138	422-FB14-4BEA-B82C-7A7C901328EA	303	*Hepatozoon musa* isolate Co1031 18S ribosomal RNA gene, partial sequence	KX880079	99.7
						HAM-1F/HPF-2R			MG758137		1679	*Hepatozoon* sp. DG1 small subunit ribosomal RNA gene, partial sequence	FJ719813	97.8
						BTF2/BTR2		*Theileria apogeana* n. sp.	MG758126	808CAD4C-D259-40E3-B929-E308D23AADBD	790	*Theileria* sp. K1 18S ribosomal RNA gene, partial sequence	JQ682879	95.8
						Nbab_1F/18SApiR			MG758116		1480	*Theileria* sp. K1 18S ribosomal RNA gene, partial sequence	JQ682879	96.5
			ITF8	D-ITF8	Seaforth, NSW	18SApiF/18SApiR		cf. Sarcocystidae sp.	MG758127	N/A	304	*Besnoitia darlingi* isolate 18SBd small subunit ribosomal RNA gene, partial sequence	MF872603	98.4
						BTF2/BTR2	Negative	N/A	N/A		N/A	N/A	N/A	N/A
						HepF300/Hep900	Positive	cf. Sarcocystidae sp.	MG758128		572	*Besnoitia besnoiti* isolate bb-CRO1 18S ribosomal RNA gene, partial sequence	KJ746531	96.5
						HEMO1/HEMO2	Negative	N/A	N/A		N/A	N/A	N/A	N/A
Rhipicephalus	Female	Dog	RSF1	D-RSF1	Torres Strait,	18SApiF/18SApiR	Positive	*Babesia canis*	MG758129		300	Uncultured *Babesia* clone seqBCV91 18S ribosomal RNA gene, partial sequence	JN717135	100

sanguineus (*n* = 2)					QLD	BTF2/BTR2		*vogeli*	MG758130		746	*Babesia canis vogeli* isolate cat 100 18S ribosomal RNA gene, partial sequence	KT323935	100
						HepF300/Hep900	Negative	N/A	N/A		N/A	N/A	N/A	N/A
						HEMO1/HEMO2								
	Larva	Dog	RSL1	D-RSL1	Katherine, NT	18SApiF/18SApiR	Positive	*Babesia canis*	MG758131		270	Uncultured *Babesia* clone seqBCV91 18S ribosomal RNA gene, partial sequence	JN717135	100
						BTF2/BTR2		*vogeli*	MG758132		746	*Babesia canis vogeli* isolate cat 100 18S ribosomal RNA gene, partial sequence	KT323935	100
						HepF300/Hep900	Negative	N/A	N/A		N/A	N/A	N/A	N/A
						HEMO1/HEMO2								

*Not applicable (N/A)

[a]The chromatogram that was obtained from Sanger sequencing of the 18S gene with the 18SApiF/18SApiR primer set was mixed, i.e. two or more fluorescent signals were observed for each nucleotide position.
