# Supplementary material for: Endemic, exotic and novel apicomplexan parasites detected during a national study of ticks from companion animals in Australia
Source: Parasit Vectors. 2018 Mar 20;11:197. doi: 10.1186/s13071-018-2775-y (PMC5859549; doi:10.1186/s13071-018-2775-y)
Supplement: Supplementary file 4 — Table S4. Pairwise genetic distance matrix of 18S sequences similarities (%) from novel piroplasms and Hepatozoon spp. described in this study. (PDF 202 kb) [file 13071_2018_2775_MOESM4_ESM.pdf]

Additional file 4: Table S4. Pairwise genetic distance matrix of *18S* sequence similarities (%) from novel piroplasms and *Hepatozoon* spp. described in this study

***18S* sequences (Accession number)**	**1**	**2**	**3**	**4**	**5**	**6**	**7**	**8**	**9**	**10**	**11**	**12**	**13**	**14**	**15**	**16**	**17**	**18**	**19**	**20**	**21**	**22**	**23**	**24**
1. *Babesia lohae* n. sp. from IHF1 long 18S (MG593272)		100	96.2	96.3	77.7	75.8	77.7	75.8	77.7	75.8	77	73.5	85.7	83.2	84.8	84.7	81.3	79.1	85.4	79.1	86	77	86	77
2. *Babesia lohae* n. sp. from IHF1 short 18S (MG593273)	100		96.2	96.7	78.4	78.4	78.4	78.4	78.4	78.4	76.2	76.2	77.8	0[a]	77.8	77.9	0	81.2	79.1	81.2	77	79.4	76.9	79.4
3. *Babesia mackerrasorum* n. sp. HspM1 long 18S (MG593271)	96.2	96.2		100	77	76	77	76	77	76	76.5	74	85.1	82.2	84.8	84.6	80.8	79.6	85	79.6	85.9	79.7	85.8	79.7
4. *Babesia mackerrasorum* n. sp. HspM1 short 18S (MG593276)	96.3	96.7	100		78.7	78.7	78.7	78.7	78.7	78.7	76.9	76.9	78.1	0	78.9	78.9	0	81.6	79.4	81.6	79.5	81.6	79.4	81.6
5. *Hepatozoon banethi* n. sp. ITF2 long 18S (MG758133)	77.7	78.4	77	78.7		100	99.8	99.7	99.8	99.7	94.8	96.1	80.7	75	82	81.7	76.3	79.8	81.5	80.1	80.7	79.3	80.8	79.3
6. *Hepatozoon banethi* n. sp. ITF2 short 18S (MG758134)	75.8	78.4	76	78.7	100		99.7	99.7	99.7	99.7	96.1	96.1	77	0	78.6	78.7	0	79.8	77.9	80.1	77	79.3	76.9	79.3
7. *Hepatozoon banethi* n. sp. ITF6 long 18S (MG758136)	77.7	78.4	77	78.7	99.8	99.7		100	99.9	100	94.8	96.4	80.7	75	81.9	81.6	76.3	79.8	81.5	79.8	80.7	79.3	80.8	79.3
8. *Hepatozoon banethi* n. sp. ITF6 short 18S (MG758135)	75.8	78.4	76	78.7	99.7	99.7	100		100	100	96.4	96.4	77	0	78.3	78.4	0	79.8	77.5	79.8	77	79.3	76.9	79.3
9. *Hepatozoon banethi* n. sp. ITF7 long 18S (MG758137)	77.7	78.4	77	78.7	99.8	99.7	99.9	100		100	94.5	96.4	80.7	75	81.9	81.6	76.3	79.8	81.5	79.8	80.7	79.3	80.8	79.3
10. *Hepatozoon banethi* n. sp. ITF7 short 18S (MG758138)	75.8	78.4	76	78.7	99.7	99.7	100	100	100		96.4	96.4	77	0	78.3	78.4	0	79.8	77.5	79.8	77	79.3	76.9	79.3
11. *Hepatozoon ewingi* n. sp. HBM1 long 18S (MG593275)	77	76.2	76.5	76.9	94.8	96.1	94.8	96.4	94.5	96.4		100	80.6	75.4	81.6	81.4	76.3	78.5	81.2	78.5	81	78.7	81.2	78.7
12. *Hepatozoon ewingi* n. sp. HBM1 short 18S (MG593274)	73.5	76.2	74	76.9	96.1	96.1	96.4	96.4	96.4	96.4	100		76.2	0	77.2	77.3	0	78.5	76.1	78.5	76.2	78.7	76.2	78.7
13. *Theileria apogeana* n. sp. ITF7 long 18S (MG758116)	85.7	77.8	85.1	78.1	80.7	77	80.7	77	80.7	77	80.6	76.2		100	92.8	92.7	89.5	97.5	97.2	97.1	95.1	92.9	95.1	92.9
14. *Theileria apogeana* n. sp. ITF7 BT2 (MG758126)	83.2	0	82.2	0	75	0	75	0	75	0	75.4	0	100		89.5	89.5	89.5	0	96.2	0	93.9	0	93.9	0
15. *Theileria palmeri* n. sp. ITF1 long 18S (MG758113)	84.8	77.8	84.8	78.9	82	78.6	81.9	78.3	81.9	78.3	81.6	77.2	92.8	89.5		100	100	92.8	93.4	93.2	93.7	95.3	93.7	95.3
16. *Theileria palmeri* n. sp. ITF6 long 18S (MG758120)	84.7	77.9	84.6	78.9	81.7	78.7	81.6	78.4	81.6	78.4	81.4	77.3	92.7	89.5	100		100	92.9	93.3	93.2	93.6	95.4	93.6	95.4
17. *Theileria palmeri* n. sp. ITF6 BT2 (MG758125)	81.3	0	80.8	0	76.3	0	76.3	0	76.3	0	76.3	0	89.5	89.5	100	100		0	90.5	0	90.6	0	90.6	0
18. *Theileria paparinii* n. sp. ITF1 short 18S (MG758112)	79.1	81.2	79.6	81.6	79.8	79.8	79.8	79.8	79.8	79.8	78.5	78.5	97.5	0	92.8	92.9	0		99.6	99.7	93.2	93.9	93.2	93.9
19. *Theileria paparinii* n. sp. ITF4 long 18S (MG758115)	85.4	79.1	85	79.4	81.5	77.9	81.5	77.5	81.5	77.5	81.2	76.1	97.2	96.2	93.4	93.3	90.5	99.6		100	95.1	92.8	95.1	92.8
20. *Theileria paparinii* n. sp. ITF4 short 18S (MG758117)	79.1	81.2	79.6	81.6	80.1	80.1	79.8	79.8	79.8	79.8	78.5	78.5	97.1	0	93.2	93.2	0	99.7	100		92.8	93.6	92.8	93.6
21. *Theileria worthingtonorum* n. sp. ITF3 long 18S (MG758114)	86	77	85.9	79.5	80.7	77	80.7	77	80.7	77	81	76.2	95.1	93.9	93.7	93.6	90.6	93.2	95.1	92.8		100	100	100
22. *Theileria worthingtonorum* n. sp. ITF3 short 18S (MG758118)	77	79.4	79.7	81.6	79.3	79.3	79.3	79.3	79.3	79.3	78.7	78.7	92.9	0	95.3	95.4	0	93.9	92.8	93.6	100		100	100
23. *Theileria worthingtonorum* n. sp. ITF5 long 18S (MG758121)	86	76.9	85.8	79.4	80.8	76.9	80.8	76.9	80.8	76.9	81.2	76.2	95.1	93.9	93.7	93.6	90.6	93.2	95.1	92.8	100	100		100
24. *Theileria worthingtonorum* n. sp. ITF5 short 18S (MG758119)	77	79.4	79.7	81.6	79.3	79.3	79.3	79.3	79.3	79.3	78.7	78.7	92.9	0	95.3	95.4	0	93.9	92.8	93.6	100	100	100	

[a]Zero indicates that there were no overlapping regions in the sequences
